# An Analysis of the United States and United Kingdom Smallpox Epidemics (1901–5) – The Special Relationship that Tested Public Health Strategies for Disease Control

**DOI:** 10.1017/mdh.2019.74

**Published:** 2020-01

**Authors:** Bernard Brabin

**Affiliations:** 1 Clinical Division, Liverpool School of Tropical Medicine, Pembroke Place, Liverpool, L3 5QA, UK; 2 Institute of Infection and Global Health, University of Liverpool, UK; 3 Global Child Health Group, Academic Medical Centre, University of Amsterdam, The Netherlands

**Keywords:** Smallpox, Maritime, Epidemic, Public health, Boston, Liverpool

## Abstract

At the end of the nineteenth century, the northern port of Liverpool had become the second largest in the United Kingdom. Fast transatlantic steamers to Boston and other American ports exploited this route, increasing the risk of maritime disease epidemics. The 1901–3 epidemic in Liverpool was the last serious smallpox outbreak in Liverpool and was probably seeded from these maritime contacts, which introduced a milder form of the disease that was more difficult to trace because of its long incubation period and occurrence of undiagnosed cases. The characteristics of these epidemics in Boston and Liverpool are described and compared with outbreaks in New York, Glasgow and London between 1900 and 1903. Public health control strategies, notably medical inspection, quarantine and vaccination, differed between the two countries and in both settings were inconsistently applied, often for commercial reasons or due to public unpopularity. As a result, smaller smallpox epidemics spread out from Liverpool until 1905. This paper analyses factors that contributed to this last serious epidemic using the historical epidemiological data available at that time. Though imperfect, these early public health strategies paved the way for better prevention of imported maritime diseases.

## 1 Introduction

A constellation of factors contributed to the pattern of smallpox outbreaks in the United States and United Kingdom at the onset of the twentieth century. This dreadful disease had occurred in sequential epidemics throughout the nineteenth century in British cities,^1^ and was largely imported from Europe.^2^ In contrast in the United States, with the exception of mild smallpox in the southern states and a few severe cases in New York City, the disease had, by 1897, entirely disappeared from the country.^3^ This changed in late 1896, following an outbreak of a very mild type of smallpox which originated in the southern states and spread over four years to northeastern cities and ports. Historical epidemiology suggests importation of smallpox from these ports to the United Kingdom, and particularly to Liverpool from Boston in 1901. The barrier of the Atlantic Ocean was now bridged by fast transatlantic steamers, shortening crossing times to fewer than six days,^4^ heightening commercial shipping interests while allowing rapid dissemination from infected sailors. The Liverpool Dock System between 1890 and 1906 was radically reconstructed, allowing intake of a greater number of larger ships from America.^5^ Liverpool expanded to become the second largest port in the United Kingdom at the turn of the century.

This paper describes the factors that contributed to the pattern of national smallpox outbreaks in the United States and United Kingdom, and specifically in the cities of New York, Boston and Liverpool, between 1901 and 1903. Reconstructing these historical epidemics using imperfect sources is challenging, and methodological limitations are considered. The primary aim is to describe public health approaches to control smallpox during epidemics in major transit ports for Atlantic shipping, and factors which influenced these efforts. Experience with different responses helped develop a more evidence-based approach to disease control and to anticipate the general public’s response to such measures. A knowledge base for disease control was growing, given experience with other maritime imported epidemic diseases, such as cholera and plague, but smallpox differed due to the availability of an effective preventive vaccine, the efficacy of which had not been fully assessed. In analysing these data, a secondary aim is to examine the evidence that smallpox cases occurring in Liverpool in 1901 and 1902 may have originated from American imported cases. Peak smallpox incidence in the United States spanned the period 1901 and 1902 and a high infection risk was channelled via ships travelling from Boston to Liverpool, where the outbreak peaked in April 1903. Outbreaks across northern and central England were temporally related to the Liverpool epidemic.

The response to the epidemics was influenced by new epidemiological approaches and public health practices in both countries, although public health recommendations differed. In the United States, national and state vaccination strategies varied, as did exemption regulations for children and adults. In both countries, local factors influenced commercial interests, variable clinical disease patterns, delayed diagnosis and quarantine practices. An improved understanding of smallpox disease epidemiology slowly emerged and contributed to eventual control and elimination through broadened international efforts.

## 2 Methodological Approaches to Reconstructing Historical Epidemiological Evidence

Extracting and interpreting late nineteenth-century information from historical medical records on smallpox in order to quantify risk factors, a standard method in modern disease epidemiology, is subject to several pitfalls. Instead of meticulous tracing and recording of known cases and their contacts, the basic assumption at that time attributed the social and domestic habits of the poor to be the principal factors spreading smallpox.^6^ Emphasis on environmental and aggregate models of health and disease suggested that microorganisms causing a specific disease were subordinate to the person’s total environment.^7^ It is true that environmental conditions are important, but by the late nineteenth century it was also realised that epidemics were caused by a specific agent. Although the organism responsible for smallpox had not been isolated, it caused a recognised disease, which evoked introduction of tighter measures to prevent its importation. This included inspecting ships, monitoring smallpox outbreaks at home and abroad, and some level of contact tracing.^8^ Europe-wide pandemics from 1870 to 1875 had led to improved vaccination strategies, with legal provisions for enforcement. These developments, the basis of modern preventive epidemiology, occurred despite inadequate understanding of the causal agent, its modes of transmission, or the relative impact of behavioural changes and social determinants.^9^

Despite such progress, the present reconstruction of historical outbreaks and examination of their risk factors is affected by several criteria which are difficult to quantify. These included: variable definitions of reported events; misdiagnoses; lack of detailed household transmission data; spatial heterogeneities; inadequate information on vaccine effectiveness – partly because of lack of reliable estimation methods; difficulties in early recognition of smallpox cases and confusion with chicken pox or measles; notification delayed until the afflicted person had been suffering for many days and the absence of explicit statistical analyses.^10^ ^,^ ^11^ Since the length of the incubation period can only be known for individuals exposed early, late reporting compromised quarantine regulations which specified a period of fourteen to sixteen days. In practice, it was based on clinical experience and limited epidemiological data. Similarly, vaccine coverage of the general population, or subgroups, was critical in order to reach a target capable of interrupting an epidemic. In the modern era, estimates of critical vaccine coverage are obtained by estimating the ’reproduction number’, which is based on the average number of secondary cases which arise from a single index case in a susceptible population in the absence of interventions. During the early twentieth century, lack of reliable statistical methods precluded measurement of vaccine effectiveness and, as a consequence, use of smallpox vaccine remained controversial. In this paper, vaccine efficacy is calculated from the data available, both in the United States and the United Kingdom. In 1903, Boston Health Department physician Dr Frank Morse pointed out that accurate smallpox records had been kept only since 1888, and were reported in the Annual Reports of the City Health Department Surgeon, as well as the Annual Reports of the Surgeon General of the Public Health, and Marine-Hospital Service of the United States. Nevertheless, this information provided no data on how many people were vaccinated or re-vaccinated out of the total population.^12^ In the United Kingdom, case numbers and locations were listed in the Annual Reports of City Medical Officers of Health, and the Metropolitan Asylums Board, and these provided crude estimates of vaccinated and allegedly vaccinated individuals, although criteria for identifying vaccine scars were unclear. Part of the analysis in this paper uses information based on these reports for the years 1901 to 1905. Monthly case notifications are available in both the United States and United Kingdom, but seasonal analysis of case fatality is limited, as available reports provide mostly annual data on deaths. The availability of these data also enables evaluation of the hypothesis that the Liverpool outbreaks originated in the United States, where disease control measures differed and may have been less efficient.

## 3 Maritime Relationships between Britain and the United States

### 3.1 Early Twentieth-Century Maritime Quarantine Regulations at British and United States Seaports

In the late nineteenth century, quarantine stations and regulations for the sanitary inspection of ships were present at many British seaports, and general sanitary arrangements were satisfactory in two-thirds of the sixty port sanitary districts.^13^ Similar arrangements were present on the Atlantic seaboard of the United States.^14^ Several diseases were cause for concern, including cholera, plague, yellow fever and smallpox.^15^ Sanitary inspection of all vessels entering British or United States ports was the main strategy available. The UK Public Health (Shipping) Act of 1885 extended the ordinary powers of local authorities granted in the 1872 Public Health Act to the Port Sanitary Authorities with respect to infectious disease.^16^ These initiatives allowed efficient intervention when vessels with infected crew or passengers entered ports.^17^ To this end, smallpox figures for countries from which other ships originated were included in reports in both countries,^18^ ^,^ ^19^ and could be used to enhance surveillance.

There was some collaboration between the two countries. The United States Assistant Surgeon, Dr Carroll Fox (b.1874), stayed in the Liverpool United States Consulate for three months in 1902 to review infection control policy and practice and the numbers of smallpox and typhus cases.^20^ He reported on six Liverpool municipal hospitals as well as on containment, refuse disposal and disinfection procedures, noting that steam disinfectors used in Liverpool were newer than those used by the Public Health Service at the quarantine station in Port Townsend, Washington. This exchange signalled some of the first international efforts to harmonise infection control practice across major sea routes for maritime transport. Yet, in September 1902, as Dr Fox completed his mission, eleven ships with infected seamen from Boston had already arrived in Liverpool, and Dr Fox failed to mention the Boston epidemic in his report.

In the last decade of the nineteenth century, the twin systems of medical inspection and quarantine were in use, but with greater emphasis on medical inspection and case isolation. The risk posed by foreigners had become more evident to the general public in the United States as migration sensitised opinions, and foreigners became a focus for quarantine policies.^21^ ^,^ ^22^ In the United Kingdom, the Merchant Shipping Act of 1894 required medical inspection of all outward bound steerage passengers and crew, when services of a medical practitioner could be obtained, on board ship or preferably before embarkation.^23^ In the Port of London, Gravesend, the Medical Officer for the Board of Trade commonly examined all persons as they proceeded along the gangway and refused them permission to proceed, if considered ill.^24^ This screening had low sensitivity, but should have identified obviously sick travellers with facial rashes, which might be a sign of active infection. As an alternative to inspection, the argument for quarantine of ships was contentious. A *Lancet* editorial commented in 1880 that it only survived because it was plausible, seductive and fitted the unreasonable demands of certain Continental powers, and that ’it was derogatory to England that she should submit to these hideously farcical detrimental proceedings’.^25^ Quarantine avoided the trouble of disinfecting and removing the sick, but was costly for trading sea ports. Moreover, unless cholera, plague or yellow fever was existent on board a vessel, there was no legal authority for detaining her on sanitary grounds. Some had advocated the inclusion of smallpox in the cholera, plague and yellow fever order, but a smallpox reservoir in the United Kingdom was assumed so its inclusion was considered inadvisable, as it would deter international trade.

### 3.2 Quarantine Stations

In Britain, quarantine stations, including some more isolated offshore establishments, had existed in the early nineteenth century. This remained the only effective measure until later in the century when contact tracing and surveillance were introduced. Port Sanitary Authorities established hospital ships in a number of locations around Britain to isolate suspected smallpox cases. In 1884, the Metropolitan Asylums Board moored three converted ships in the Thames to serve as a floating hospital,^26^ primarily for indigenous cases arising during the 1884–5 London smallpox epidemic.^27^ By 1899, the Infectious Diseases Notification Act required compulsory notification of infections, by which time smallpox was the focus of attention.^28^ In Liverpool in 1874, the Local Government Board, under the Public Health Act, permanently constituted the Liverpool Council with powers to inspect vessels on arrival and to appoint a quarantine station in the River Mersey where vessels could anchor. A quarantine station already existed at Hoyle Lake, an offshore area enclosed by sandbanks on the outer Mersey Estuary, which provided accommodation for infected patients, particularly cholera cases.^29^ As the estuary began silting up and Liverpool port expanded, a land-based Port Sanitary isolation hospital was built in 1875 at New Ferry, isolated from the public, and with ship access via a long wooden jetty.^30^

**Figure 1:**
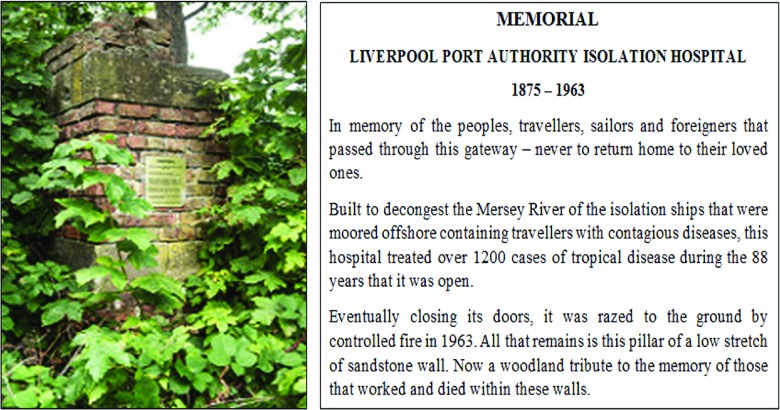
Liverpool Port Sanitary Hospital Commemorative plaque.

Procedures were in place for ship fumigation, cleaning and painting of vessels, disinfection of clothing, and vaccination of passengers and seamen, although often seamen refused vaccination.^31^ ^,^ ^32^ This task was immense, given the number of ships passing through the Port of Liverpool. More than 1200 cases of tropical diseases were admitted to the Port Sanitary Hospital in the period 1875–1963, including cholera, leprosy and smallpox. Some infectious disease patients were still treated as ordinary patients in other Liverpool hospitals, including smallpox cases, which were later transferred to New Ferry.^33^ As early as 1891, New Ferry was declared unnecessary. Instead, long-haul ships proceeded directly to the Pier Head entrance of each Liverpool dock to answer questions on quarantine, a concession much appreciated by ship owners.^34^ In 1896, quarantine was discontinued and officially replaced by medical inspection,^35^ although in practice quarantine remained, but at the discretion of the Local Government Board rather than as a national policy.^36^ New Ferry simply acted as an isolation hospital for eighty-eight years and was officially razed to the ground in 1963. A commemorative plaque records its historic role and location and is the last physical reminder of a Port Sanitary hospital in the United Kingdom (Figure 1). Even today, quarantine is exerted only in exceptional circumstances.

In the United States, maritime quarantine was initially a state service but was transferred to a national Public Health Service between the 1880s and the 1920s. The Marine Hospital, dedicated to the care of ill and disabled seamen in the United States Merchant Marines, the US Coast Guard and other federal beneficiaries, eventually evolved into the Public Health Service Commissioned Corps.^37^ Quarantine stations were established,^38^ and a prescribed protocol followed, based on the vessel’s sanitary history and presence of infected or deceased crew or passengers. Disease detection led to active disinfection and fumigation of ships and isolation of passengers. When a case of smallpox was diagnosed on board, crew and passengers were expected to spend fourteen days in quarantine, although breaches of recommended policies were often made, especially for travellers in first and second class.^39^

## 4 Factors Leading up to the Boston 1901–2 Smallpox Epidemic in the United States

Smallpox, when diagnosed, was reported and case fatalities were recorded across the country. Dr Charles Value Chapin (1856–1941), an American pioneer in public health research and practice, and Health Superintendent (1884–1932) for Providence, Rhode Island, compiled a detailed outline of smallpox in the United States between 1895 and 1912.^40^ By 1897, with the exception of mild smallpox in the South and a few severe cases in New York City, the disease had disappeared from the country. During 1896, cases of mild smallpox in Florida began to spread and within a period of about four years cases were detected all over the country.^41^ The rate of dispersion was exponential because the infection was mild, which meant that patients remained active, and contagious, in their communities and cases were under-reported.^42^ By 1900 this wave of infection had reached northeastern cities, and by 1901 had carried smallpox to every state and territory in the Union. There had been an epidemic in New York City in late 1899, preceding that in Boston in 1901.^43^ ^,^ ^44^ The main culprit was the milder strain (*Variola minor*), with had a death rate of 2 to 6% among unvaccinated individuals, which was considerably lower than with the *Variola Major* strain.^45^ *Variola major* nonetheless remained present in several American cities, particularly in the northeast.

**Figure 2:**
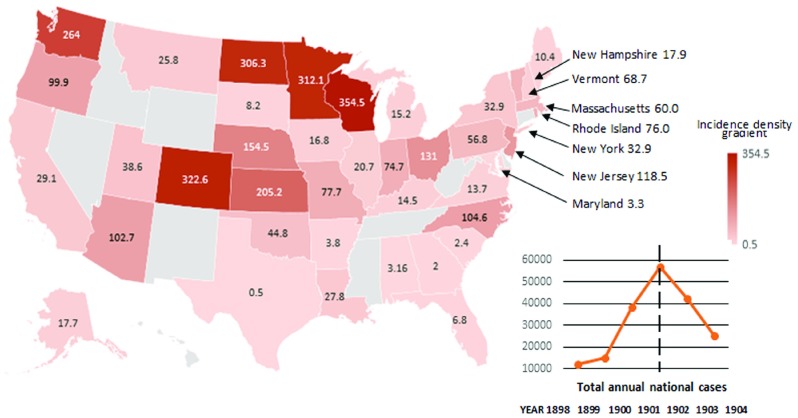
United States smallpox incidence per 100 000 population per annum, 28 June 1901–27 July 1902. *Sources:* National Population Census data of 1900 [note 47]; Annual Report of State Smallpox Notifications of the Surgeon General of the Public Health and Marine-Hospital Service for 1901 [note 48].

Disease notification was incomplete in some cities and rural areas, and some states omitted returns.^46^ Incidence for individual states returning notifications can be estimated using 1900 National Population Census data and the 1901 Annual Report of state smallpox notifications of the Surgeon General of the Public Health and Marine-Hospital Service. ^47^ ^,^ 48 Figure 2 shows these estimates by state between 28 June 1901 and 27 July 1902 per 100 000 population. By 1901, as the epidemic spread to northern states, incidence rapidly declined in southern states, ranging from less than one per  population in Texas, to more than 300 cases per  population in North Dakota, Minnesota and Wisconsin. Annual smallpox notifications peaked nationally in 1901 at 56 857 cases (Figure 2). In Massachusetts, where the 1901 Boston epidemic occurred, annual incidence for that year was sixty per  population. In New York City, between 1901 and 1902, 3480 smallpox cases were reported, with 720 deaths (incidence approximately 100 cases per 100 000 population).^49^

The Boston epidemic commenced in May 1901 in a large factory.^50^ Cases were initially mild but by May 1901 severe cases began to appear. Their source was not known. The Health Department thought a letter received by a family from infected relatives living through the New York epidemic was the cause.^51^ In the outbreak, twelve cases within forty-eight hours were admitted to hospital, and despite control measures, cases increased to thirty in September, forty-nine in October, 195 in November, and 201 in December 1901.^52^ By the end of October 1902, new cases of smallpox had appeared in nearly every section of the city with the epidemic continuing into 1903. In total, there were 1596 cases (period incidence 284 per 100 000 population) with 270 deaths (17%).^53^ A smaller concurrent smallpox epidemic occurred in the adjoining city of Cambridge, Boston’s close neighbour across the Charles River.^54^ The nationally prominent senior public health officer in Boston, and Health Department Chairman, Dr Samuel Holmes Durgin (1839–1931), initially played down the epidemic, referring to it as only ’a flurry of cases’ and a minor storm that would pass.^55^ Durgin advised that schools should remain open and insisted that immediate vaccination rather than quarantine was the most effective control strategy. Many leading physicians in Boston preferred vaccination to sanitation and quarantine, and were somewhat indifferent to public anxiety over contagion.^56^ Durgin was blamed for the continued spread of smallpox, not least because he had allowed the Health Department physicians who treated smallpox patients to mingle with and expose the public without taking precautions.^57^ The cost of quarantine weighed heavily on officials and the controversy received front-page press coverage.^58^

The difference in severity of cases between epidemics warrants further examination. Characteristics of this epidemic can be gleaned from the clinical records of 243 patients consecutively admitted to the Southampton Street smallpox hospital in Boston.^59^ Some hospital cases were caused by the *Variola major* form of smallpox, but these were not necessarily representative, as many attacks were mild.^60^ Smallpox has an incubation period of approximately seven to seventeen days, clinical onset leading to headache and backache, fever and malaise during a three-day pre-eruptive period before appearance of a skin rash. Hospital cases presented with varying severity, but 47% were the milder form of the disease, and most occurred in previously vaccinated persons. Full recovery took weeks, with most deaths occurring as a result of unrecognised cases of smallpox in the city and suburban districts going about from place to place, with many unvaccinated people being exposed to infection.^61^ In the initial febrile phase (two to four days), individuals would be infective, but infected seafarers, or their contacts, may not have developed symptoms until nearly three weeks later. Individuals incubating the disease could readily depart on transatlantic ships and act as disease vectors.^62^

The Boston epidemic coincided with a smaller smallpox epidemic (after accounting for the difference in population size) in London, commencing in June 1901 and lasting until January 1903, with 9484 notified cases.^63^ There is no evidence that the London and Boston outbreaks were connected, despite their similar timing of onset. The daily surveillance returns to the Metropolitan Health Board for the 1901–3 London epidemic indicated upwards of twenty different centres of infection. The origin of the disease for the first two patients could not be traced and, so far as was known, no cases arose from contact with them. Two foci at the end of June identified a Parisian male who infected four people, including a laundry worker who infected nine other contacts.^64^ In August, several other cases, whose contact source could be traced, seemed unconnected. Preceding both these epidemics was an outbreak in Glasgow, which began in April 1900 and lasted until July 1901 (1786 notified cases), with a recrudescent period between January and May 1902 (469 notified cases).^65^ The Glasgow epidemic terminated prior to the onset of the Boston epidemic and the two are unlikely to have been directly related. A small epidemic occurred in Dublin in 1903 with fewer than 250 cases and fewer than forty deaths,^66^ which resulted from indigenous transmission with an index case from Glasgow. Vessels from Boston did not sail via Dublin (see Figure 4 in next section).

## 5 Transatlantic Sailings Transmitting Smallpox from the Eastern United States to Liverpool

In the nineteenth century, thousands of emigrants from the British Isles left from Liverpool Port. Packet lines sailed regularly from 1818, and in 1822 smallpox was transmitted from Liverpool to Baltimore on board the ship *Palla*s.^67^ Demand for North American timber and cotton to meet British industrial expansion led to well-established transatlantic links. British manufactured products provided a useful return cargo. Steamships started to replace sail after the 1860s and the average voyage time was reduced to as few as six days.^68^ The city of Liverpool was largely dependent upon the sea for its commercial prosperity. Several steamship lines at the turn of the century were competing for transatlantic passengers to Boston from Liverpool, and American cattle ships departing from Boston traded regularly with the City.^69^ Schedules of ships arriving in Liverpool from Boston for different steamship companies resulted in multiple arrivals each week, and round trip transits took less than a month. In 1902, tonnage entering and leaving the River Mersey amounted to the colossal total of 29 000 000 tons, with 214 000 emigrants.^70^

Prior to the 1901–3 epidemic, smallpox was imported on eight known occasions to Liverpool in 1900, the most important being that of the *SS New England*, which arrived with nineteen cases on board. This ship left Boston on 1 February, arriving in Liverpool on 30 March. On leaving Boston with 525 passengers and 268 crew, including fifty-five clergymen and many elderly people, it travelled to the Mediterranean.^71^ On 11 March, prior to arriving in Constantinople, a male death occurred after presenting with petechial skin haemorrhages, which were attributed to liver atrophy. The body was buried at sea. By 21 March, twelve other people were sick, including eight crew, two of whom were employed in the laundry, and two who had been assigned to seal the dead man’s body in a casket. By 23 March, other passengers were sick with presumed malarial fever and biliousness, and passenger deaths were reported following visits to Jaffa and Naples. The Captain’s log only records smallpox after 22 March, and it is unclear why he sought smallpox vaccination for himself on the 11 March while docked in Constantinople. The ship’s doctor had no previous experience of smallpox. At Naples, all remaining 500 passengers were peremptorily disembarked and given tourist tickets, while the vessel left port and sailed for Liverpool without communicating with the port on the nature of this disease outbreak. The United States Marine Hospital *Fortnightly Gazette* reported that a number of these passengers fell ill with smallpox at Naples and in other places in Italy and France.^72^ Three persons subsequently developed the disease in Liverpool on dates which indicated it was contracted prior to disinfection of the ship, which occurred later at Liverpool. On 8 July 1900, the *SS Ivernia* from Boston also landed a single smallpox case at Queenstown while *en route* to Liverpool.

**Figure 3:**
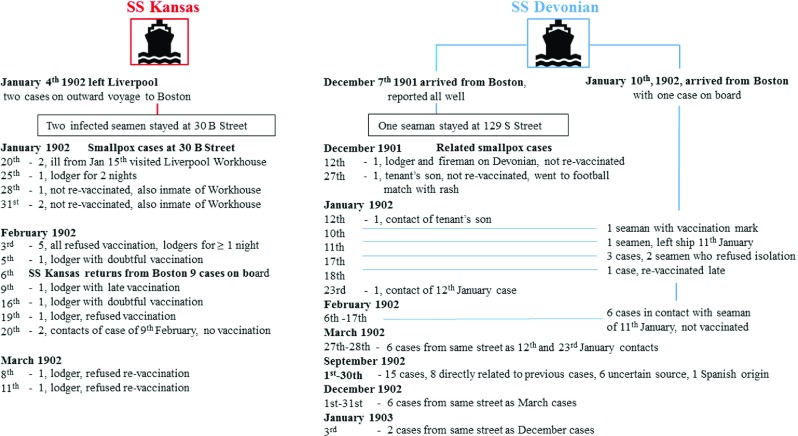
Neighbourhood smallpox transmission linked to imported maritime cases from Boston. *Sources:* Port Sanitary Hospital Archives [note 30]; Annual Report on Health of the City of Liverpool during 1902 [note 73].

Competitive, fast transatlantic passenger and mail steamers were efficient disease vectors. Figure 3 illustrates three examples of transit involving two ships arriving during the initial phase of the Liverpool epidemic.^73^ These ships left Boston during the period of peak prevalence during the epidemic of 1901–3.

**Figure 4:**
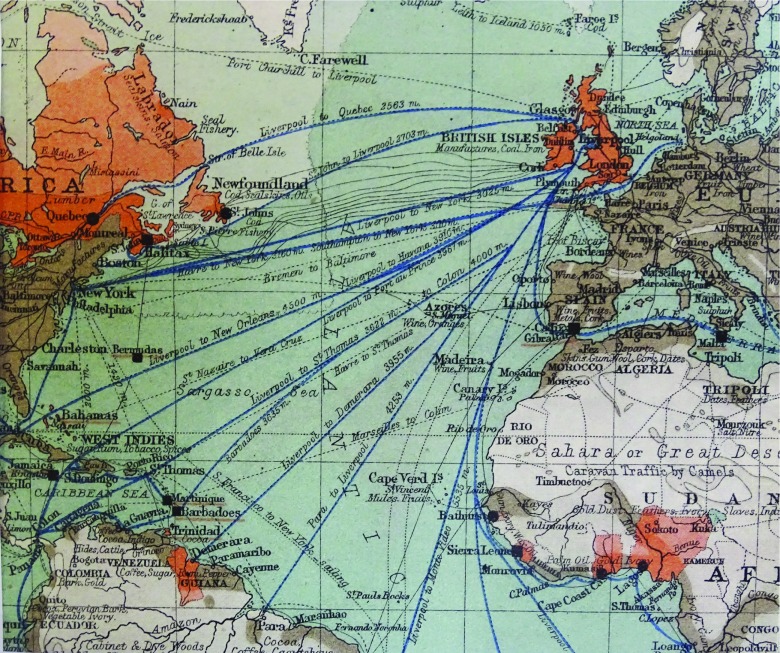
Commercial map showing the transatlantic trade connections of the Port of Liverpool in 1903. *Source:* Appendix, City of Liverpool. Handbook Compiled for the Congress of the Royal Institute of Public Health, edited by E.W. Hope (Liverpool: Lee and Nightingale, Printers, 1903). Map production Spottiswoode and Co., Ltd., Liverpool. Commercially developed: red, British Empire; grey, other countries. Detail of transatlantic section from a world map.

Dates in Figure 3 indicate when notified and not when the illness began. The *SS Kansas* arrived in Boston on 15 January 1902 following one smallpox death at sea. The ship was quarantined but the vessel was allowed to leave for its return trip to Liverpool after only six days. When it arrived back in Liverpool, nine clinical cases were identified on arrival and transferred to the Port Sanitary Hospital at New Ferry, one of whom died.^74^ The crew had been vaccinated before leaving Boston but some were already incubating the disease. The Boston Health Department Chairman, Dr Samuel Durgin, received criticism in the press for allowing the ship to depart after so few days of quarantine.^75^ At a legislative hearing on an anti-vaccine bill, Durgin responded as follows to a critical heckler: ’I hold the public health of Boston in one hand and its commerce in the other’.^76^ Commercial interests in Boston were influential factors affecting public health regulations, at least in this instance. The Health Department deliberately gave the outbreak a low profile to prevent an unwanted scare. Press releases stated that alarm was needless and claimed smallpox has never been epidemic in the city.^77^

The *SS Devonian* carried infected seamen on separate occasions in early December, January and February. With the mild type illness, diagnosis was unclear until medical advice on skin spots was sought.^78^ A further maritime case was identified on the *SS Campania* arriving from New York on 5 April, on a ship holding the fastest transatlantic crossing time.^79^ Multiple secondary Liverpool cases arose from these infected seamen, especially through local lodging houses and contact with workhouse inmates. Seven different ships were carrying infected passengers, and the *SS Kansas* imported nineteen cases from Boston that were admitted to the New Ferry hospital. When the ship sailed from Liverpool on 4 January 1902, two crew were treated at sea, one of whom died. Late acquisition of smallpox explained these cases. Upon reaching Boston, as many as twenty men were put ashore and all cattlemen were taken to the quarantine station and revaccinated.^80^ Returning to Liverpool after leaving Boston, the *SS Kansas* put back to New York and landed several further crew suffering from smallpox. Later arriving at Liverpool on 6 February, nine convalescents and two contacts were identified and removed to the Port Sanitary Hospital.^81^ Most of the crew were revaccinated and some were kept under close observation.

**Figure 5:**
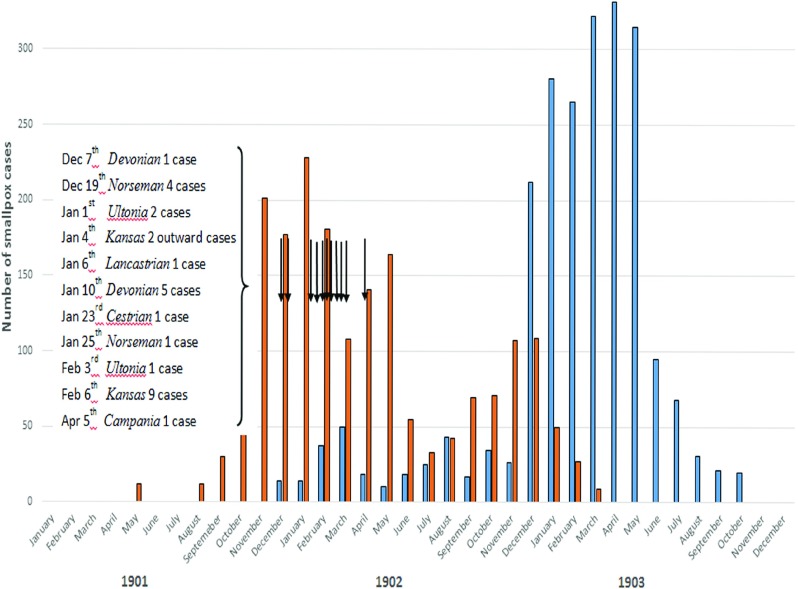
Periodicity of Boston and Liverpool epidemics and dates of transatlantic ship sailings. *Sources:* [reference notes, 43, 50, 53, 68, 69].

Figure 4 shows the commercial trade connections of the Port of Liverpool and the multiple potential routes for inward transmission of smallpox in 1903.^82^ Vessels from Liverpool travelling west to Boston discharged first at St Johns, Newfoundland, and Halifax, Nova Scotia. On the return journey, these vessels often travelled directly to Liverpool. Between 1901 and 1902, only a single case of smallpox was reported in Halifax, affecting a seaman on a schooner leaving Gloucester, north of Boston.^83^ No deaths from smallpox were reported in St Johns during this period,^84^ which suggests westward transmission of smallpox from Liverpool was negligible. The number of maritime cases of smallpox landed from vessels in the Port of Liverpool between 1900 and 1904 is shown in Table 1 in relation to port origin and case fatality. The total number of identified cases arriving from Boston represents 27% of all maritime cases. The months involved are shown in detail in Figure 5, to illustrate occurrence in Liverpool and steamship arrivals from Boston with diagnosed smallpox infected crew or passengers. Another 27% of importations arrived from New York and Baltimore in 1903 and 1904. Other incoming vessels from outside the United States were also responsible for importations of cases or suspected cases of smallpox (Table 1), but the majority of single-country origin was from the United States.

**Table 1:** Number of maritime cases of smallpox landed from vessels in Port of Liverpool 1900–4 in relation to United States origin and case fatality. *Sources: Annual Reports on the Health of the City of Liverpool during 1900–4*; Liverpool Smallpox Register, Wirral Archives [reference notes 30, 93].

Year	Cases from ships	Cases from ships arriving	Annual	Annual
	arriving from Boston	from other locations	case total^b^	case fatality
	 (%)^a^	 (%)	 (%)	 (%)
1900	1 (3.7)^c^	26^d^	156	23 (14.7)
1901	5 (21.7)^e^	18	37	6 (16.2)
1902	20 (44.4)^f^	25	560	20 (3.6)
1903	4 (16.6)^g^	20	1720	141 (8.2)
1904	4 (66.6)^h^	2	27	2 (7.4)
Total	34 (27.2)	91	2500	192 (7.7)

a Percentage of Boston and East Coast United States transits of all Liverpool maritime cases for that year.

b Annual number of cases for the city of Liverpool.

c Single case landed at Queenstown, Ireland.

d Includes nineteen cases on ship from Boston via the Mediterranean; one case on ship from New York.

e Transported on two different ships.

f Transported on seven different ships.

g Four cases from New York. Sixteen different ships brought cases, or suspected cases, of smallpox.

h Four cases from Baltimore.

## 6 Maritime and Non-Maritime Spread of Smallpox in the United Kingdom 1901–5

The connexion between seaports and smallpox had been initially observed in England in the epidemic of 1870–2, when Liverpool and London were the first places to feel the effects of the continental outbreaks associated with the Franco-Prussian war. In Liverpool, smallpox had been introduced by Spanish sailors.^85^ Liverpool experienced almost 2000 deaths in the 1871 epidemic, but these numbers decreased with 685 deaths during the 1876–7 epidemic and 34 deaths in the smaller 1881 epidemic.^86^

**Figure 6:**
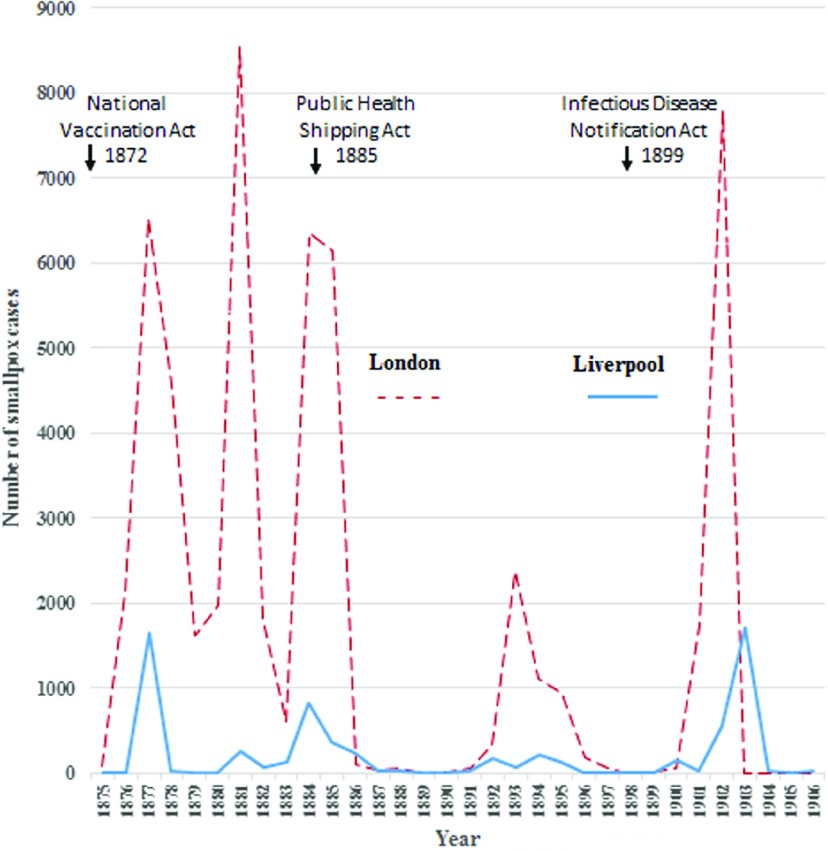
Periodicity of London and Liverpool smallpox epidemics between 1875 and 1905. *Sources:* [reference notes, 27, 63, 93].

The 1901 Liverpool outbreak was the last major smallpox epidemic in this city. Its magnitude was comparable to earlier 1876–8 outbreaks, as shown in Figure 6. Four Liverpool epidemics had occurred between 1875 and 1896, which exactly corresponded temporally with the London epidemics, spanning both the same years and having identical epidemic periods. This suggests indigenous transmission between these cities. In contrast, the Liverpool epidemic of 1901 commenced twelve months after onset of the London epidemic and was characterised by milder infections and close association with imported maritime cases. Yet in London during 1902, of ninety-three smallpox cases treated in their Port Sanitary Hospital, only one was an imported infection from New York, admitted on 12 April from the *SS Minnehaha*.^87^ Other cases were internally transferred to London, mostly from British Ports – particularly Newcastle, with additional single importations from Spanish, German and Dutch ports, as well as India and South Africa.^88^

**Figure 7:**
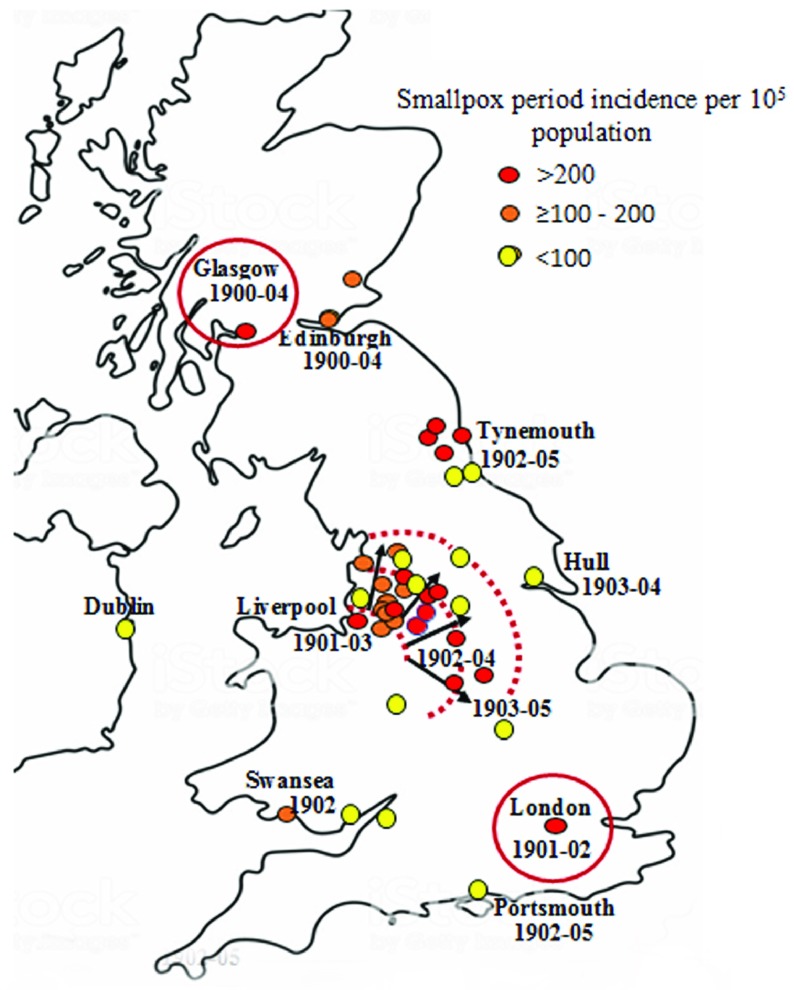
United Kingdom spatial smallpox period incidence per  population 1900–5. *Sources:* McVail, Table II, 6 [note 49]; United Kingdom National Census, 1901 [note 99].

John Christie McVail (1849–1926), Medical Officer of Health for Stirling and Dumbarton in Scotland, and a leading advocate of smallpox vaccination in the early twentieth century, suggested that smallpox was no longer indigenous in the United Kingdom and insisted that epidemic outbreaks were imported.^89^ He tabulated provincial smallpox outbreaks in English and Welsh cities and towns between 1902 and 1905.^90^ Incidence estimates per capita can be derived from these case numbers using the 1901 United Kingdom National Census. These are listed in Table 2, and their spatial dispersion mapped in Figure 7. Case fatality estimates are also tabulated for the same locations.

**Table 2:** United Kingdom smallpox period incidence and case fatality 1900–5. *Sources:* McVail, Table II, 6 [note 49]; Martin, Table II, 19 [*Journal of Hygiene*, 34, 1(1934)]; UK Census, 1901 [note 39].

Location	Period in	Cases	1901 Census	Period incidence	Deaths	Case fatality
	years^a^		population	per 		(%)
Lancashire & North-West England						
Liverpool	1901–3	2280	684,958	333	160	7.0
Stockport	1902–4	159	78,897	201	15	9.4
Oldham	1902–3	413	137,246	301	32	7.7
Chadderton	1902–5	144	24,000	600	5	3.5
Wigan	1902–3	70	60764	115	1	1.4
Blackburn	1902–3	141	127,626	110	5	3.5
Salford	1902–4	262	220,957	119	12	4.6
Manchester	1902–4	563	543,872	103	33	5.9
Warrington	1903	86	64,242	134	4	4.7
Macclesfield	1903–4	69	37,500	184	5	7.2
Preston	1904–5	172	112,989	152	8	4.7
Bradford	1901	28	279,767	10	0	0.0
St Helens	1902–5	66	84,410	78	3	4.5
ALL	1901–5	4453	2,457,228	181	283	6.3
Yorkshire & Pennines						
Ossett Union	1902–3	519	12,903	4022	61	11.8
Heckmondwike	1904	91	9500	958	5	5.5
Dewsbury	1904	552	28,060	1967	57	10.3
Leeds	1902–5	690	428,968	161	35	5.1
Halifax	1903	141	104,936	134	6	4.3
York	1902–4	39	77,914	50	7	17.9
Batley	1904	103	128,712	80	6	5.8
ALL	1902–5	2135	790,993	270	172	8.0
Central England						
Leicester	1902–4	731	211,579	345	30	4.1
Derby	1903–4	255	105,912	241	5	2.0
Nottingham	1903–5	479	239,743	200	17	3.5
Sheffield	1902–4	141	380,793	37	5	3.5
Northampton	1902–3	44	87,021	51	9	20.5
Birmingham	1902–5	364	522,204	70	17	4.7
ALL	1902–5	2014	1,547,252	130	83	4.1
North-East England						
Tynemouth	1902–5	328	51,366	639	17	5.2
South Shields	1902–5	272	97,263	280	14	5.1
Chester-le-Street	1903–4	106	34,000	312	6	5.7
Newcastle	1903–5	628	215,328	294	28	4.5
Durham	1902	35	419,782	8	1	2.9
Sunderland	1902–3	66	146,077	45	4	6.1
Hull	1903–4	141	240,259	77	8	5.4
ALL	1902–5	1576	1,204,075	131	78	4.9
South Wales and South-West England						
Swansea	1902	187	94,537	198	187	17.1
Cardiff	1901–5	96	164,333	58	5	5.2
Portsmouth	1902–5	20	188,133	11	1	5.0
Bristol	1903–5	125	328,945	38	1	3.2
ALL	1902–5	428	479,948	89	39	9.1
London						
Greater London	1901–2	9484	6,226,494	152 (203)^b^	1540	16.2
Scotland and Ireland						
Glasgow	1900–4	3413^c^	762,000	448	371	10.9
Edinburgh	1900–4	191	303,638	63	16	8.4
Dundee	1903–4	175	154,734	113	12	6.9
Rest of Scotland	1900–4	2844	3,251,731	87	235	8.3
Dublin (hospital)	1903–4	243	448,000	54	33	13.6

a Annual periods may not include all months of the year dependent on month outbreak commenced or resolved.

b Brackets is incidence estimate based on inner city London population alone.

c Includes some cases from beyond city boundaries.

The Liverpool focus is distinct from those in Glasgow, London, Edinburgh, Tynemouth, Hull and South Wales, which are all ports, but which did not have regular scheduled transatlantic links with eastern United States seaports. In Southampton, only two cases of smallpox were reported on vessels bound for the port in 1902.^91^ Smallpox outbreak distribution shows temporal dispersion across northwest and central England between the years 1902 and 1904. The dispersion pattern implicates Liverpool as the primary focus, possibly with discrete sequential transmission across the northwest during these years. In these outbreaks, case fatality was low or very low (Table 2) and in the northwest averaged 6.3%. This mortality was much lower than in the 1901 London epidemic (21.6%), suggesting that infection was mostly due to a different milder strain, consistent with transmission mainly from the Liverpool focus where a mild strain of *Variola minor* was dominant. Chapin had considered it highly probable that the mild type of smallpox was carried to England from Boston in 1902 and during the following years.^92^ Case fatality during the epidemic decreased from around 16% in 1901 to 8% in 1903.^93^ As all cases were hospitalised, this may have caused a bias towards observing severe or fatal cases.^94^ More than half the national deaths from smallpox in 1902 occurred in the northwest of England, either in Lancashire or in the contiguous West Riding of Yorkshire. In both cases, about four-fifths of the mortality took place in the course of the first half of the year, with the majority of deaths in the city of Liverpool.^95^

The first infected Liverpool resident was identified on 12 December 1901 (Figure 3), from a lodging house ’infected’ by an imported maritime case.^96^ Between December 1901 and November 1902, there were three minor epidemics, followed by a major epidemic between December 1902 and December 1903. The minor epidemics corresponded temporally to the period of initial importation of Boston cases (Figure 3). The onset of the ensuing major epidemic corresponded with the second peak in prevalence in Boston. Indigenous Liverpool cases who had some direct or indirect contact with Boston seamen can be identified up until January 1903. Between October 1902 and December 1903, 108 households reported cases which accommodated sailors or mariners.^97^ Imported cases from other East Coast American ports were recorded in 1903 (Table 1). Smallpox was circulating in other British ports, and internal transmission via English coastal shipping cannot be ruled out, as these ports did not have Port Sanitary Stations and were not subject to the same surveillance as in Liverpool.

**Figure 8:**
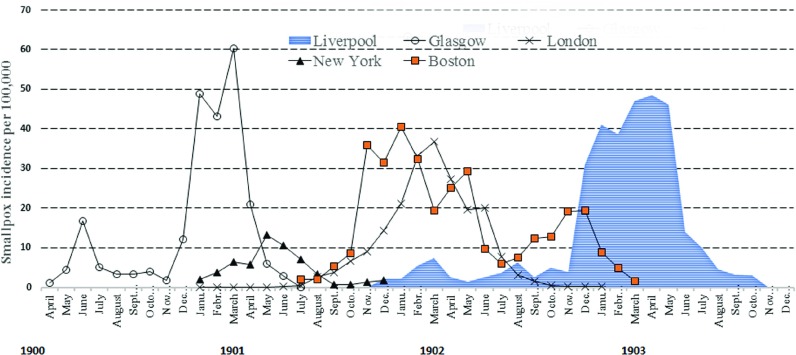
Comparative smallpox incidence 1900–3 per 100 000 population for Liverpool, Glasgow, London, New York and Boston. *Sources:* [reference notes 3, 53, 66, 93]; W.J. Martin, ’The epidemic curve of smallpox’, *Journal of Hygiene (London)*, 34, 1 (1934), Table II, p. 19; United States National Census 1900 [note 47]; United Kingdom National Census 1901, [note 99].

Figure 8 shows the temporal associations of the New York, Boston, Glasgow, London and Liverpool epidemics between 1900 and 1903. Incidence is estimated using population denominators from the United States 1900 census and British 1901 census.^98^ ^,^ ^99^ All epidemic periods (time of onset to time of resolution) shown in Figure 8 are less than two years. The Liverpool and Boston epidemics covered almost identical periods (Liverpool, twenty-four months; Boston, twenty-three months), although the shape and distribution of the monthly totals are dissimilar. Peak incidence per 100 000 population was very similar in Boston and Liverpool. Marked left-sided skewness is evident for the Liverpool and Glasgow epidemics, suggesting low early transmission rates. The source of the Glasgow outbreak was attributed to severe smallpox in a seaman on board the *SS Hispania*, which arrived from Bombay.^100^ Left-sided skewness was not evident in the London outbreaks in which maritime importations were considered less critical. This conclusion is supported by Hardy, who has commented that for previous London epidemics:

‘*In 1881, although smallpox was epidemic in the metropolis, the cases occurring in the port were few in number and “quite isolated”. There were six imported cases. The smallpox epidemics occurring in London in 1876, 1881, and 1884 cannot with certainty be traced to* *infection from abroad: it is possible that the strain introduced in 1870 was working itself out with diminishing virulence, and that the port sanitary authorities were successful in preventing its refreshment from abroad*,’^101^ and:

‘*In December 1876 the Port Medical Officer reported that although smallpox was widely epidemic in London, it had so far manifested itself in the floating population to an infinitesimal extent.*’^102^

The resolution of the Liverpool epidemic was rapid, with a marked fall in incidence in June 1903, which compares to a much slower resolution in Boston over several months. As described below, this may relate to differences between the two cities in the effectiveness of the public health response, epidemiological approaches to disease control, as well as the lower vaccine uptake in Boston and staggered introductions of vaccine delivery. Case fatality in unvaccinated persons in Boston was very similar to that in Liverpool (Table 3), whereas fatalities in unvaccinated persons in the London and Glasgow epidemics were double that of Boston and Liverpool. It is therefore likely this was predominantly a milder illness consistent with a *Variola minor* strain in Boston and Liverpool, whereas the higher case fatality in unvaccinated cases in London and Glasgow is consistent with *Variola major* as the primary source of infection.

**Table 3:** Case fatality, smallpox vaccine efficacy and coverage during Liverpool, London, Glasgow and Boston epidemics between 1901 and 1903.

City	Allegedly			Admittedly			Total			Vaccine efficacy %	% cases	% population protected
	unvaccinated			vaccinated						(95% confidence interval)^a^	vaccinated	(95% confidence interval)^b^
	Cases	Deaths	%	Cases	Deaths	%	Cases	Deaths	%			
Liverpool^c^
Fazakerley Hospital	119	27	22.7	571	15	2.6	690	42	6.1	90.8 (82.1–95.3)	82.7	75.1 (67.9–78.8)
Park Hill Hospital	277	65	23.5	1063	35	3.3	1340	100	7.5	88.9 (82.8–92.8)	79.3	70.5 (65.7–73.6)
London^d^	194	98	50.5	760	108	14.2	954	206	21.6	83.8 (77.0–88.5)	79.7	57.1 (48.9–63.4)
Glasgow^e^	122	63	51.6	1642	150	9.1	1764	213	12.8	90.6 (86.1–93.6)	93.1	84.3 (80.2–87.1)
Boston^f^	842	188	22.3	754	82	10.9	1596	270	16.9	57.6 (43.8–67.9)	47.2	27.2 (25.2–32.0)

a Percentage reduction in case fatality in the vaccinated compared to unvaccinated group. Estimated from: attack rate unvaccinated minus attack rate vaccinated, divided by attack rate vaccinated  100. An alternative equivalent formulation of vaccine efficacy is [1 minus relative risk]  100, where relative risk is risk of developing the disease (or case fatality in this table) for vaccinated compared to unvaccinated people.

b Calculated from [vaccine efficacy  percentage cases vaccinated], on assumption percentage cases vaccinated is indicative of population vaccine coverage.

c *Source:* Edward W. Hope. *Annual Report on the Health of the City of Liverpool in 1903.* C. Tinling and Co., Printing Contractors, Liverpool, 1904, pp. 35, 39 [note 73].

d *Source:* The Jenner Society. *The London Epidemic of Smallpox*. London, 1901, p. 1. Analysis of epidemic data for London for 1901.

e *Source:* John C. McVail. ’Smallpox in Glasgow, 1900–2.’ *British Medical Journal*, (1902): 40–3. Total cases mostly excludes children under five years.

f *Source:* M.R. Albert, K.G. Ostheimer, J.G. Breman. ’The last smallpox epidemic in Boston and the vaccination controversy, 1901–3.’ *New England Journal of Medicine*, 344, 5 (2001): 375–8 [note 53].

## 7 Other Factors Influencing the Course of the Boston and Liverpool Epidemics

### 7.1 Public Health Measures and Quarantine

It is now known that smallpox can have a long incubation period (up to twenty-two days),^103^ and a relatively low infectious rate without close direct contact, which would cause it to spread slowly within or between communities.^104^ In 1902, the incubation phase was less well defined, and containment strategies for asymptomatic contacts who were potentially incubating the disease varied. In both Boston and Liverpool, emphasis on land was on search and containment. Outbreaks were aborted by vaccination around each focus, but contacts were not necessarily placed in quarantine. In Boston, houses were kept under observation by medical inspectors for fourteen days, with removal of secondary cases, but contacts were free to travel, even if they refused vaccination. The absence of quarantine led to heated exchanges published in the Cambridge Chronicle, in which the mayor and the State Medical Board doctor recommended quarantine, whereas the Board of Health opposed it.^105^ ^,^ ^106^ Health authorities is some towns recommended quarantine for immigrants or migrant workers. Men living in inexpensive rooming houses were ordered to be vaccinated by the Boston Board of Health,^107^ but it allowed settled inhabitants their freedom. Quarantine was outrageously expensive and health departments that relied on vaccination alone justified this as efficiency and fiscal responsibility.^108^ Whether to quarantine or vaccinate thus involved social, economic and cultural considerations.

In the United Kingdom, the Scottish obstetrician Sir James Young Simpson (1811–70) wrote an influential outline on smallpox prevention based on household isolation policies. Its departure point was early case notification, quarantine of infected patients, vaccination of carers, hygienic cleaning of everything in contact with the patient, and strict disinfection and bedding procedures.^109^ Households were to be fumigated by burning sulphur for five hours, stripping and burning wallpaper, and spraying the interior with mercury perchloride solution, with up to thirty-four houses treated daily.^110^ These practices were regularly followed in Liverpool during the 1901 epidemic, but isolation of contacts was less straightforward and opinions were divided on whether smallpox was transmitted in the prodromal phase of the illness when the patient was febrile but had no rash – the main indicator for virus transmissibility. Ships with onboard crew in a vessel infected with smallpox were not routinely quarantined, since the Public Health Act of 1896 did not require this, and detention for longer than thirty-six hours was considered unreasonable.^111^ In fact, apparently healthy passengers could disembark from their ships without knowing they were transmitters. Cattlemen from Boston sometimes sold their return passage tickets, remaining in Liverpool lodging houses, and often in particular streets and the poorer districts.^112^ This, together with misdiagnoses due to the uncharacteristic rashes related to milder illness, meant that containment and contact tracing were only partially successful. Epidemic proportions of infection first occurred in Liverpool in areas such as Everton, having insanitary housing awaiting demolition, many of which were overcrowded, and the highest case fatality.^113^ Homeless tramps were also blamed for spreading infection in Liverpool,^114^ as were vagrants during the Boston epidemic.^115^

### 7.2 Views on Modes of Transmission that Affected the Public Health Response

In the late nineteenth century, the mode of transmission of smallpox was not well understood. It was considered intensely infectious, arising potentially from aerosol spread, infected fomites and direct contact with the patient, their clothing or belongings. In industrialised countries, it was considered a ‘winter disease’^116^ because even short-term epidemics clearly exhibited an annual peak in January. An analysis by Sir Leonard Rogers (1868–1962) of the mild type of smallpox in England and Wales from 1921 to 1927 showed peak seasonal prevalence, closely related to low absolute humidity and lower temperatures.^117^ Peak prevalence in Boston in 1901 was in January, and in Liverpool covered the months January to March in 1903. A similar monthly pattern was observed in Glasgow and London (Figure 8), although the possible seasonal pattern remains unexplained for this period.

The smallpox register for Liverpool queried whether two cases had occurred from contact with foreign mail.^118^ Sir William Osler (1849–1919), a young Canadian physician in 1876, had disinfected his letters when he had smallpox,^119^ and in his popular 1892 general textbook of medicine he supported the fomite transmission theory. The *British Medical Journal* in 1901 reported smallpox transmission to Nottingham by Mormon mail from Salt Lake City,^120^ where it was unlawful to compel vaccination.^121^ Transatlantic steamers were the only means of mail transportation from North America, but there is no evidence of disinfection procedures involving mail.

A major concern was infection of communities living in proximity to isolation hospitals, including the New Ferry hospital. Confusion over aerial transmission, and in particular controversy over spread of disease from hospitals, was a major concern,^122^ and was a reason the Liverpool outbreak was studied in great detail.^123^ This issue was investigated by Dr Richard J. Reece and reported in a memorandum to the Liverpool Medical Officer of Health in 1905.^124^ Following detailed statistical analysis, Reece concluded that smallpox risk was disproportionately high in areas nearer to the hospital during periods in which it was receiving acute smallpox cases. This conclusion was queried by Dr Edward William Hope (1856–1951), the Medical Officer of Health for Liverpool, who listed ten points of contention, noting that high numbers of cases also occurred around hospitals not receiving smallpox cases, and for one hospital, cases occurred before the receiving hospital was opened.^125^ Although disputed,^126^ Reece defended his conclusions, considering that aerial convection probably operated in the case of each Liverpool hospital.^127^ ^,^ ^128^ Sixty years after these exchanges, community concerns about neighbourhood smallpox transmission in the vicinity of the Port Sanitary Hospital at New Ferry were still being voiced following incidence of residential infections within a quarter mile radius of the hospital.^129^ ^,^ ^130^ ^,^ ^131^ ^,^ ^132^ This concern was also expressed by the Chief Public Health Inspector at the time, who implicated fly transmission.^133^ An airborne outbreak of smallpox in a German hospital was reported in 1970, which demonstrated the local features believed to be of importance in this unusual pattern of transmission.^134^

Modern understanding has improved knowledge of infection risk, although the minimum infectious dose of smallpox remains unknown, ie. how many virus particles an individual needs to inhale to become infected.^135^ The probability of airborne tiny particles (less than a thousandth of a millimetre in size) from surfaces seems low, because the smaller the particle the more avidly it adheres, reducing the risk of fomite transmission.^136^ This issue was reassessed as late as 1978 during the legal case surrounding the last case of smallpox in the United Kingdom, when long-distance airborne spread of smallpox from a laboratory was dismissed as improbable.^137^ A recent meta-analysis calculated that when index cases were quickly identified and control established early, the reproduction rate to secondary generations was low, which is consistent with limited aerial transmission. When index cases remained unidentified a large increase in the median initial reproduction rate occurred.^138^ ^,^ ^139^ In the Liverpool epidemic, a crude upper estimate of the reproduction rate (defined as the expected number of new infected hosts that an infectious host will produce in a large randomly mixed population of susceptible individuals), was up to twenty, based on index and secondary cases, suggesting late case recognition with a high reproduction rate.^140^ This rate may be overestimated, as contact status was not always available and tertiary or later generation cases may have been included. There may be an interval of two to three weeks between each generation of cases and even during the transmission season an index case rarely infected as many as five persons.^141^ Failure to instigate effective case isolation and contact tracing would have prolonged the Liverpool epidemic.

### 7.3 Vaccine Efficacy and Uptake

Table 3 compares case fatalities in several cities in relation to vaccination status. Vaccine efficacy is estimated for each site. Vaccine efficacy in this context is the percentage reduction in case fatality in the vaccinated compared to the unvaccinated group. Such estimates were not reported at the time of the United States and United Kingdom outbreaks, as an accepted formula for its calculation had not yet been proposed. Statistical methods to evaluate how likely it is that any observed difference between the groups arose by chance were first proposed in 1900 by Karl Pearson (1857–1936), founder of the modern field of statistics, using the chi square test.^142^ It was not until 1915 that epidemiologists derived measures related to efficacy.^143^ Publications and reports on smallpox epidemics in the early twentieth century provided only descriptive statistics with listings of cases and deaths.^144^ ^,^ ^145^ ^,^ ^146^

From the estimates of vaccine efficacy in Table 3, it can be concluded that it was considerably lower in Boston than Liverpool, reflected in the higher death rate of vaccinated cases in Boston. Mortality in unvaccinated cases was similar in both cities. Vaccine efficacy estimates for the London and Glasgow outbreaks were much higher than that for Boston. The proportion of the hospitalised population vaccinated in Boston was only 27%, which was less than half that for the United Kingdom cities. Vaccine uptake in the United States was irregular,^147^ and by the 1930s four states still had laws prohibiting compulsory vaccination, twenty-nine had no vaccine laws, six provided for a local option and four had made it compulsory.^148^ In Boston, state law by 1855 required that children be vaccinated to enter public school but adult vaccination was voluntary. Yet enforcement was problematic and in 1902 there was uncertainty on how many people were vaccinated in Boston, as no state or federal agency compiled data. In England, infant vaccination had been compulsory since 1853, with substantial penalties for non-compliance in 1902.^149^ In 1898, an amendment to the Public Health Act allowed conscientious objection, although early in the twentieth century fewer than 200 000 exemptions were granted, equivalent to about 25% of all births.^150^ In 1901, coverage of infants in England due to be vaccinated was approximately 78%.^151^

In both countries, community-wide vaccination and revaccination campaigns were started during the epidemics. In Boston, uptake was impaired by the unpopularity of the vaccine, side effects seemingly a greater problem than mild smallpox.^152^ Massachusetts vaccination law also penalised vaccination refusal with a $5 fine. In January 1902, legislation was proposed by anti-vaccination supporters, including some physicians, to repeal state compulsory vaccination laws.^153^ The anti-vaccination campaign undermined public acceptance of Health Department authority, as it disputed the utility of vaccination on medical grounds as well as based on restriction of civil liberties. The well-recognised Boston physicians Dr Caroline Eliza Hastings (1841–1922) and Dr Sarah Newcomb Merrick (b.1844) sparred with Dr Durgin, the Chair of Public Health, disputing the merits and safety of the vaccine.^154^ This debate captured the ideological interface between anti-vaccinationists and public health. A focal point was the British Anti-vaccination League, which had strong links to New England.^155^ In 1905, the United States Supreme Court eventually upheld the right that states could mandate compulsory vaccine laws under their police powers.^156^ The story of these judicial proceedings highlights the critical role for medical authorities to gain public trust from a diverse audience in ensuring the success of a public health campaign. In England, public concern about vaccination led to the introduction of the conscience clause in the new vaccination act.^157^ In Boston, intermittent vaccine availability from private and public sources, doubts about the efficacy of vaccine lymph, concerns about complications, including documented contaminated vaccine transmission of syphilis and tetanus, compounded public anxiety so much that public confidence plummeted.^158^ There were staggered introductions of vaccinations programmes, with closures and reopening of vaccine stations in Boston and Cambridge.^159^ It is likely these factors contributed to the length of the post-peak epidemic period that occurred in Boston.

In Liverpool, widespread smallpox vaccination was prioritised and the benefits of vaccination were strongly promoted locally.^160^ The question of effectiveness arose due to cases occurring in known vaccinees, and it was understood that primary vaccination alone would not prevent severe epidemics.^161^ Some individuals used the conscientious objection clause in the 1898 Public Health Act to refuse vaccination. During the epidemic, the Medical Officer of Health for Liverpool supported an initiative to repeal this clause,^162^ stating that 460 000 people remained susceptible out of a population of 750 000 (61%).^163^ This is lower than the estimate in Table 3, derived from hospital data. With coverage thus reduced, medical officers also had to rely on public health surveillance and containment, with selective vaccination of contacts.^164^

The reasons for higher vaccine efficacy in Liverpool compared to Boston are unknown. This may relate to vaccine potency or storage conditions, differing pre-epidemic population immunity, or herd immunity,^165^ and/or strain variations in smallpox virus.^166^ Boston and Liverpool authorities produced different vaccines derived from varying strains. Badcock’s lymph was generally used in England,^167^ and Mulford’s pure lymph vaccine in the East Coast United States.^168^ Bacterial contamination became a problem in the United States in 1901–2 when the demand for vaccine was great, perhaps indicating haste in production.^169^ Vaccine production was poorly regulated, with competition between several private firms. In 1895, Walter Reed (1851–1902), the eminent United States Army pathologist and bacteriologist, had presented a paper to the District of Colombia Medical Society titled ‘What Credence Should Be Given to the Statements of Those Who Claim to Furnish Vaccine Free of Bacteria’.^170^ His answer, after examining vaccine needle points, was ‘none at all’.^171^ By early 1902, the medical profession realised that national licensing was required, as it was contradictory to require compulsory vaccination without product safety. An Act of Congress was signed by President Theodore Roosevelt on 1 July 1902, requiring manufacturers to comply with production standards, after which four firms went out of business.^172^

Vaccine virus deteriorates if not cooled in transit, and transport in baggage cars in the United States had to avoid proximity to steam coils which affected vaccine quality.^173^ In Liverpool, Dr William Hanna, assistant Medical Officer of Health for Liverpool, noted that below-freezing temperatures could easily be obtained on board most larger sea vessels, and a suitable supply of efficient lymph (ie. vaccine) could be stored for months and be ready in an emergency.^174^ Lack of available vaccine aboard oceanic vessels remained an important limitation.

In theory, vaccination not only diminishes the susceptibility of vaccinated individuals but also reduces the degree and duration of infectiousness.^175^ In Liverpool, only a few vaccinated children less than five years of age developed smallpox, indicating protection following infant vaccination that lasted longer than five years.^176^ An assessment of vaccination during the Liverpool epidemic was presented almost a decade later by Dr Hanna in a book entitled *Studies in Smallpox and Vaccination*.^177^ Post exposure vaccination of contacts early in the incubation period was recommended. This strategy of selective ring vaccination of contacts living proximate to cases which was used in Liverpool was adopted during the World Health Organisation smallpox eradication campaign of the 1970s. Despite challenges by the Anti-vaccination League,^178^ Liverpool remained one of the best-vaccinated cities in the country.^179^

### 7.4 Press Responses

Two Liverpool city newspapers printed regular press communications, with occasional notices in the local Wirral Birkenhead press, which covered the New Ferry Sanitary Hospital area. The press was responsible for reporting accurately the local position. Detailed summaries of medical presentations by Dr Edward Hope, held at The Liverpool Medical Institute, were printed for public consumption, and included conclusions of local City Council meetings.^180^ Newspapers promoted vaccination and emphasised epidemic surveillance by reporting case numbers, which must have reassured the general public. It was less alarming to attribute new cases to maritime sources than to discover a local source of contamination.^181^ Local opposition remained, and prominent individuals opposed to vaccination published their views in the national press, including George Bernard Shaw (1856–1950).^182^ Spurious claims appeared in *The Liverpool Echo* for use of alternative remedies, such as a ‘curative syrup’ (Mother Seigels) in times of epidemic, that would keep the whole body strong, avoiding enfeeblement by indigestion, anaemia, blood disorders and lack of stamina necessary to resist contagion.^183^ A qualification noted, ‘Mother Siegel’s Syrup does not cure smallpox, but will purify the blood, build up the system and give increased strength to resist contagion and disease’.

Multiple press news releases appeared in the *Boston Globe* and *Cambridge Chronicle* as the outbreak developed. The Globe reported in December 1901 that the outbreak had abated, was at no time serious, and that the public seemed to have been needlessly alarmed with largely imaginary dangers.^184^ Disputes on conflicting medical advice were published, and prosecutions for refusing vaccination, as well as the official advice of the Massachusetts Anti-compulsory Vaccination Society,^185^ together with illustrations of the public campaign to promote vaccine uptake.^186^ As in Liverpool, advertisements encouraged people to seek protection from other means, including ‘Radam’s microbe killer’ that prevented and cured every contagious disease,^187^ and Lifebuoy Soap that prevented smallpox.^188^ Howard’s Health Company cures were advertised in a journal started in November 1901 by the anti-vaccination campaigner Dr Immanuel Pfeiffer of Boston,^189^ who became infamous in February 1902 when, unvaccinated, he contracted smallpox after visiting Boston’s smallpox hospital on a dare from Dr Durgin, Boston’s Board of Health Director.^190^

## 8 The Demise of Smallpox

The data presented above support the view that in the late nineteenth and early twentieth century, smallpox had become a disease primarily introduced to the north of England from abroad by merchant seamen or by travellers. Sometimes the disease was detected onboard ship, but inadequate public health measures lead to occurrence of many missed cases. Between 1904 and 1916, only 144 further cases (six deaths) occurred in the United Kingdom, dwindling to two cases in the last three years.^191^ National smallpox control was effectively achieved in the succeeding decade. In England and Wales between 1935 and 1952 smallpox was imported on thirty-one occasions, spreading to the population twenty-five times.^192^ Maritime transmission to Liverpool occurred sporadically after 1903 and the last recorded smallpox case at the New Ferry Sanitary Hospital was a child disembarked from the *SS Cilicia* in April 1950, a P & O steamer mostly engaged in the India run.^193^ In Europe between 1950 and 1971, acquisition of *Variola minor* from infected ship crews or officials occurred in 5% of all reported cases (31/652) during that period.^194^ The danger of introduction of ship-borne smallpox into the United Kingdom was emphasised by McGregor in 1942, who still stressed everything should be done to protect passengers and crew, and so minimise the risk to Britain.^195^ Yet in the twentieth century, a valid vaccination certificate was not required for travellers to the UK by sea from India, Pakistan, Burma or Nigeria, or countries important as virus reservoirs. The emphasis switched to diagnosis by ships’ doctors and immediate vaccination of contacts onboard became the normal procedure. It was hoped that late vaccination would protect contacts, identify them as exposed and allow arising cases of smallpox to be quickly recognised.^196^ Marsden emphasised quarantine of ships, as practised in other countries, following incidents on the *SS Tuscania* in Glasgow in 1929, and *SS Cathay* in the Port of London in 1938.^197^ Dixon illustrated this with the example of the *SS Mooltan*, when contacts were quarantined for only four to five days and were then allowed free travel, leading to an outbreak which required an immense public health control effort.^198^ Longer ship quarantine periods would have greatly reduced this risk to the local population, but was not required by law. In almost the last outbreak in the UK in 1962, an initial haematological diagnosis of *P.vivax* malaria in a child whose subsequent death was attributed to staphylococcal septicaemia,was later realised to be smallpox.^199^ The pathologist who completed the post-mortem was unvaccinated and subsequently died of confluent smallpox. In the United States, *Variola minor* became the dominant form. In 1906, so far as can be learned from the United States Public Health Department’s records, severe smallpox cases were scarce, occurring in only nine of 12 503 reported cases.^200^ During the 1920s, nearly 400 000 cases were reported, with very low case fatality, and during the next decade few American states mandated vaccination and in 1971 routine childhood vaccination was discontinued.^201^

## 9 Conclusions

Smallpox was regularly transmitted across the Atlantic after its arrival from tropical Africa in the fifteenth century.^202^ Many other diseases failed to make the transfer for lack of suitable vectors.^203^ With the advent of rapid transatlantic steamers after 1860 American–United Kingdom transmission was guaranteed. Common factors across the cities of Boston and Liverpool, and late recognition of new infections in persons incubating the disease, facilitated the occurrence of large epidemics of smallpox in these two cities. Transatlantic passage of infection corresponded temporally with the period of peak national incidence in the United States. This high infection risk was most likely channelled by maritime passage to Liverpool in 1901. Despite communications in 1901 between staff at the Liverpool Port Authority and the United States Public Health Service, there is no record of heightened maritime surveillance in either city or country.

Dr Edward William Hope, the Liverpool Medical Officer of Health during the epidemic, was essentially the counterpart of Dr Samuel Holmes Durgin, Head of the Public Health Department in Boston, who was considered one of the greatest public health physicians in nineteenth-century America.^204^ Both steered development of effective control measures, through surveillance and screening, isolation, quarantine, contact tracing and selective vaccination around cases, now known as ’ring vaccination’. Both contended with anti-vaccinationists, which were especially vehement in Boston. The public health principles they implemented were later used by the global smallpox eradication programme.^205^ Post-exposure smallpox vaccination can be critical in limiting transmission, and historical analysis of the Liverpool data suggests long-lasting protective effects of smallpox vaccination.^206^ This approach, adopted in Liverpool by the Port Authority with maritime cases, helps explain the city’s success in its early control of the epidemic and the very rapid fall in incidence in 1903. Dr Hope’s work established Liverpool as an international public health authority, pre-dating the wider global effort to eradicate smallpox.^207^ This contrasts with the much more gradual fall in incidence in Boston over several months where vaccine coverage and efficacy was much lower. Later probable transmission beyond Liverpool led to wide urban dispersion in northern cities and towns between 1902 and 1904. After 1905, control activities in the United Kingdom effectively reduced cases to minimal numbers,^208^ whereas in the United States epidemics continued up until the late 1920s, when the country had the highest number of cases of any in the world except for India.^209^ The United States was the least-vaccinated country for smallpox of any in the world.^210^

The maritime origins of infection transported by record-breaking transatlantic liners of that time reflects the influence of commercial shipping interests requiring rapid turnaround times. The reluctance to implement strict quarantine, perhaps especially in Boston, was partly dictated by cost and commercial considerations. There is much to suggest that the Liverpool smallpox epidemic originated in Boston, but this is perhaps less important than other conclusions. The interchange is a reminder of the greatly magnified risks with modern air transport of unrecognised infections combined with a high priority for commercial efficiency. This historical event is an unusual example of special ties forged between the United States and the United Kingdom, through disease transmission rather than diplomacy.

## Footnotes

1.

Charles Creighton , A History of Epidemics in Britain, Volume Two 1666–1893 First edition 1894 (London: Frank Kass and Co. Ltd., 1965), 582–601 and 604–19.

2.

Donald R. Hopkins , Princes and Peasants, Smallpox in History (Chicago, IL: University of Chicago Press, 1983), 87–96.

3.

Charles Value Chapin , ‘Variation in Type of Infectious Disease as Shown by the History of Smallpox in the United States 1895–1912’, Journal of Infectious Diseases, 13 (1913), 171–196.

4.

P.J. Hugill , World Trade since 1431 (Baltimore, MD: Johns Hopkins University Press, 1993), 128.

5.

William Farrer and J. Brownbill (eds), A History of the County of Lancaster, Volume 4 (London, 1911), 41–43. British History Online http://www.british-history.ac.uk/vch/lancs.

6.

Anne Hardy , The Epidemic Streets. Infectious Disease and the Rise of Preventive Medicine 1856–1900 (Oxford: Clarendon Press, 1993), 134 and 145.

7.

Charles E. Rosenberg , Explaining Epidemics and Other Studies in the History of Medicine (Cambridge: Cambridge University Press, 1992), 299–301.

8.

Alexander Mercer , Infections, Chronic Disease, and the Epidemiological Transition: A New Perspective (Rochester, NY: University of Rochester Press, 2014), 69.

9.

S. Del Valle , H. Hethcote , J.M. Hyman and C. Castillo-Chavez , ‘Effects of Behavioural Changes in a Smallpox Attack Model’, Mathematical Biosciences, 195, 2 (2005), 228–251.1591366710.1016/j.mbs.2005.03.006

10.

Hiroshi Nishiura , Stefan O. Brockmann and Martin Eichner , ‘Extracting Key Information from Historical Data to Quantify the Transmission Dynamics of Smallpox’, Theoretical Biology and Medical Modelling, 5 (2008), 20 doi:10.1186/1742-4682-5-20.18715509PMC2538509

11.

Karen Wallach , The Antivaccine Heresy: Jacobson v. Massachusetts and the Troubled History of Compulsory Vaccination in the United States *(Rochester Studies in Medical History)*, (Rochester, NY: University of Rochester Press/Boydell and Brewer, 2015), 70–73, 253. Provides an extensive list of references covering the period 1900 to 1902 on difficulties of smallpox diagnosis in the United States.

12.

*Ibid*., 16.

13.

Anne Hardy , ‘Smallpox in London: Factors in the Decline of the Disease in the Nineteenth Century’, Medical History, 27 (1983), 128.10.1017/s0025727300042599PMC11392986306365

14.

Howard Markel , ‘A Gate to the City: The Baltimore Quarantine Station, 1918–28’, Public Health Reports, 110, 2 (1995), 218–219.7631002PMC1382106

15.

D.S. Barnes , ‘Cargo, ”Infection,” and the Logic of Quarantine in the Nineteenth Century’, Bulletin of the History of Medicine, 88 (2014), 75–101.2476980310.1353/bhm.2014.0018

16.
Public Health (Shipping) Act (1885), 48 & 49, and Public Health Act (1872), 75 & 76 Vict. c. 79, sec. 3, 20.

17.

J.C. Burne , ‘The Long Reach Hospital Ships and Miss Willis’, Proceedings of the Royal Society of Medicine, 66 (1973), 1017–1021.458632910.1177/003591577306601020PMC1645620

18.

*Annual Report of the Surgeon General of the Public Health and Marine-Hospital Service of the United States, Fiscal Year 1902* (Washington, DC: Government Printing Office, 1903), 303–3.

19.

*Annual Report of the Surgeon General of the Public Health and Marine-Hospital Service of the United States, Fiscal Year 1904* (Washington, DC: Government Printing Office, 1905), 100.

20.

*Annual Report of the Surgeon General of the Public Health and Marine-Hospital Service of the United States, Fiscal Year 1903* (Washington, DC: Government Printing Office, 1904), 192–3. He later won recognition for the identification of a quirk in the flea population of San Francisco, California, and helped prevent a wider outbreak of plague which had been infecting the city population since 1900. *Public Health Reports*, 25 September 1908, quoted in David K. Randall, *Black Death at the Golden Gate: The race to save America from the bubonic plague* (New York: W.W. Norton and Company, 2019), 210–11.

21.
Barnes, *op. cit.* (note 15).

22.

Alan M. Kraut , Silent Travelers: Germs, Genes, and the ’Immigrant Menace’ (Baltimore, MA: Johns Hopkins University Press, 1995), 50–77.

23.
Port of London Sanitary Committee. Annual Report of the Medical Officer of Health to 31 December 1902. May 1903, 23–5.

24.

*Ibid.*, Appendix H, 77–91.

25.

*The Lancet*, editorial, 1 (1880), 687–9.

26.
Burne, *op. cit.* (note 17).

27.

*London Metropolitan Asylums Board Annual Report 1900* (London: M. Corquadale and Co. Ltd., 1901).

28.
Infectious Diseases Notification Act (1899), 62 & 63 Vict. c. 8.

29.

Thomas Herbert Bickerton , Medical History of Liverpool from the Earliest Days to the Year 1920 (London: John Murray, 1936), 188.

30.
Port Sanitary Hospital, New Ferry, City of Liverpool Smallpox Register, Wirral Archives, Cheshire Lines Building Centre, Birkenhead, Wirral.

31.
Hardy, *op. cit.* (note 13), 122–3, 129.

32.
William Hanna, *Report on Marine Hygiene: Being Suggestions for Improvements in the Sanitary Arrangements and Appliances on Shipboard.*Liverpool Port Sanitary Authority (Liverpool: C. Tinling and Co. Ltd., 1917).

33.
Liverpool and Emigration in the Nineteenth and Twentieth Centuries. National Museums Liverpool, Maritime Archives and Library, Information Sheet 64.

34.

John Booker , Maritime Quarantine. The British Expansion, 1650–1900 (Hampshire, UK: Ashgate Publishing Ltd, 2007), 547.

35.
The Public Health Act 1896 (Statute 59 and 60, c19) made provisions with respect to epidemic, endemic and infectious diseases and repealed the 1825 Quarantine Act (Statute 6 Geo iv, c78), and sections in other acts in which quarantine was mentioned. The Act united for the first time the sanitary arrangements of the United Kingdom.

36.
Booker, *op. cit.* (note 34), 549–50.

37.

*United States Public Health and Marine-Hospital Service Annual Report,*1905, 221.

38.
Markel, *op. cit.* (note 14).

39.

Michael Willrich , Pox. An American History (New York: The Penguin Press, 2011), 219.

40.
Chapin, *op. cit.* (note 3), 186.

41.
Hopkins, *op. cit.* (note 2), 288.

42.

Gareth Williams , Angel of Death. The Story of Smallpox (Hampshire, UK: Palgrave Macmillan, 2010), 331–332.

43.
Chapin, *op. cit.* (note 3), 186.

44.
Willrich, *op. cit.* (note 39), 41–74, 167.

45.

*Thirty-First Annual Report of the Health Department of the City of Boston for the Year 1902* (Boston: City of Boston Printing Department, 1903), 36.

46.

*Ibid.*, 172.

47.
United States National Census 1900. 12th Census Population. United States Federal Census Records, www.Censusrecords.com.

48.

*Annual Report of the Surgeon General of the Public Health and Marine-Hospital Service of the United States, Fiscal Year 1901* (Washington DC: Government Printing Office, 1902), 319–44, 579–82.

49.

John Christie McVail , Half a Century of Smallpox and Vaccination *(Milroy Lectures)*, (Edinburgh: Edinburgh, E. & S. Livingstone, 1919), 10.

50.

*Thirtieth Annual Report of the Health Department of the City of Boston for the Year 1901* (Boston: City of Boston Printing Department, 1902), 43.

51.
Chapin, *op. cit.* (note 3), 186.

52.

*Thirtieth Boston Annual Health Department Report*, *op. cit.* (note 50), 44.

53.

Michael Albert , Kristen Ostheimer and Joel Breman , ‘The Last Smallpox Epidemic in Boston and the Vaccination Controversy, 1901–1903’, New England Journal of Medicine, 344, 5 (2001), 375–378.1117217210.1056/NEJM200102013440511

54.
Wallach, *op. cit.* (note 11), chapter three, ’The 1901–2 smallpox epidemic in Boston and Cambridge’, 75–8.

55.

*Ibid.*, 59.

56.

*Ibid.*, 68–9.

57.

*Ibid.*, 152–3.

58.
‘Three Strong Letters against Quarantine, The Board of Health’s Opinion. Dr Brough’s Opinion’, *Cambridge Chronicle*, 19 July (1896).

59.

Michael R. Albert , Kristen G. Ostheimer , David J. Liewehr , Seth M. Steinberg and Joel G. Breman , ‘Smallpox Manifestations during the Boston Epidemic of 1901–3’, Annals of Internal Medicine, 137, 12 (2002), 993–1000.1248471510.7326/0003-4819-137-12-200212170-00013

60.
Wallach, *op. cit.* (note 11), 69.

61.

*Thirty-first Boston Annual Health Department Report*, *op. cit.* (note 45), 36.

62.
Albert, *op. cit.* (note 59), 375.

63.

*Metropolitan Asylums Board Annual Report 1901, Sixteenth Report of the Statistical Committee* (London: McCorquadale & Co. Ltd., 1902).

64.

*Ibid.*, 108.

65.
Archibald Kerr Chalmers, ‘Smallpox, 1900–2’, *Corporation of Glasgow Report* (West Nile Street, Glasgow: Robert Anderson, Printer, 1902), 8–10.

66.
Detailed Annual Reports of the Registrar-General (Ireland), for 1902 (39th), 1903 (40th), 1904 (41st) containing numbers and causes of deaths registered in Ireland for that year (Dublin: His Majesty’s Stationery Office, Cahill and Co., 1903, 1904, 1905).

67.

Cyril William Dixon , Smallpox (London: Churchill Press, 1962), 198.

68.
‘The American Mails. Performances Outward and Homeward, Cunarders at the Front’, *The Liverpool Post*, 11 April (1900).

69.

Edward William Hope , Annual Report on the Health of the City of Liverpool during 1901 (Liverpool: C. Tinling and Co., Printing Contractors, 1902), 39.

70.
Thomas Clarke, ‘Introductory Address to the Section on Port Sanitary Administration, Royal Institute of Public Health Congress, Liverpool, July 1903’, *Journal of State Medicine*, 11, 8 (1903): 454–61.

71.
Port of Liverpool, *Annual Report of the Medical Officer of Health to the Port Sanitary Authority for the Year 1900* (Liverpool: C. Tinling and Co., Printing Contractors, 1901), 16–17.

72.

*Ibid.*, 17.

73.

Edward William Hope , Annual Report on the Health of the City of Liverpool during 1902 (Liverpool: C. Tinling and Co., Printing Contractors, 1903), 26–51.

74.
Port Sanitary Hospital, *op. cit.* (note 30). These men had different occupations, including baker, engineers, firemen, carpenter, sailor and boatswain.

75.
‘Eight Cases on Board’, *The Boston Globe*, 7 February (1902), 4.

76.
Wallach, *op. cit.* (note 11), chapter three, ‘The 1901–2 smallpox epidemic in Boston and Cambridge’, 61.

77.
‘Alarm Was Needless. Smallpox Has Never Been Epidemic Here. Less than Two Cases in Every 3000 of the Population of Boston’, *The Boston Globe*, 15 December (1901).

78.
‘Smallpox in Liverpool, a Fresh Case Reported Today. Forty-two Cases in the City’, *The Liverpool Echo*, 17 February (1902), 5.

79.
‘The American Mails, Performances Outward and Homeward. Cunarders to the Front’, *The Liverpool Daily Post*, 11 April (1900), 3.

80.
Hope, 1903, *op. cit.* (note 73).

81.
‘The Smallpox, Liverpool, no Fresh Cases, Infected Cattlemen Removed to Hospital’, *The Liverpool Daily Post*, 8 February (1902), 6.

82.
City of Liverpool, in E.W. Hope (ed.) *Handbook Compiled for the Royal Institute of Public Health, Liverpool Congress, 15–21 July 1903* (Liverpool: Lee and Nightingale, Printers).

83.

Alvey A. Adee , ‘A Case of Smallpox on Schooner *Thalia* at Halifax’, Public Health Reports, 16 (1901), 2176.

84.
St Johns, Newfoundland. Register of Deaths St Johns City District, Book 3 (1901–2), 333–9.

85.
Hardy, *op. cit.* (note 13), 128 and 132.

86.

*Ibid.*, 128.

87.
Port of London Sanitary Committee, *Annual Report of the Medical Officer of Health to 31 December 1902*, May 1903, appendix H, 83.

88.

*Ibid.*, appendix H, pp. 77–91.

89.
McVail, *op. cit.* (note 49), 8, 26.

90.

*Ibid.*, p. 6 (Table II).

91.
Southampton 1902 *Public Health Report*, 26.

92.
Chapin, *op. cit.* (note 3).

93.

*Annual Reports on the Health of the City of Liverpool during 1900 to 1904* (Liverpool: C. Tinling and Co., Printing Contractors, 1901). Estimates compiled from figures available from Annual Health Reports produced by Dr E.W. Hope, Medical Officer of Health for Liverpool, for the years 1900 to 1904, and published in the years 1901, 1902, 1903, 1904 and 1905.

94.

Martin Eichner , ‘Analysis of Historical Data Suggests Long-lasting Protective Effects of Smallpox Vaccination’, American Journal of Epidemiology, 158 (2003), 717–723.1456166010.1093/aje/kwg225

95.

*Sixty-sixth Annual Report of the Registrar-General of Births, Deaths, and Marriages in England and Wales (1903)* (London: His Majesty’s Stationery Office, Darling and Son, Ltd., 1905), xiviii.

96.

*Report by Dr Richard J. Reece to the Local Government Board on Smallpox and Smallpox Hospitals at Liverpool, 1902–3* (London: His Majesty’s Stationary Office, Darling and Son Ltd, 1905), 10, https://search.wellcomelibrary.org/iii/encore/record/CRb2136004.

97.

Edward William Hope , Annual Report on the Health of the City of Liverpool during 1903 (Liverpool: C. Tinling and Co., Printing Contractors, 1904), 32.

98.
United States National Census, 1900, *op. cit.* (note 47).

99.
United Kingdom National Archives, 1901 Census.

100.
Chalmers, *op. cit.* (note 65), 9.

101.
Hardy, *op. cit.* (note 13), 132, and *Half-Yearly Report of the Medical Officer* (London: Port of London, June 1881), 4.

102.

*Ibid.*, p. 132, and *Half-Yearly Report of the Medical Officer* (London: Port of London, December 1876), 9.

103.
Nishiura, *op. cit.* (note 10). The 99% upper confidence limit of the incubation period is 22.2 days, suggesting that quarantine should last twenty-three days.

104.

James A. Yorke , Neal Nathanson , Giulo Pianigiani and John Martin , ‘Seasonality and the Requirements for Perpetuation and Eradication of Viruses in Populations’, American Journal of Epidemiology, 109, 2 (1979), 103–123.21844610.1093/oxfordjournals.aje.a112666

105.
Wallach, *op. cit.* (note 11), 152–3.

106.
‘Mayer McNamee Scores a Point’, *Cambridge Chronicle*, 12 July (1902), 1; ’Smallpox Scourge’, *Cambridge Chronicle*, 21 June (1902); ‘Dr A.W. Bowman takes Issue with Other Physicians and the Board of Health’, *Cambridge Chronicle*, 9 August (1902), 6.

107.
Albert *et al.*, *op. cit.* (note 59), 375–6.

108.
Wallach, *op. cit.* (note 11), 68–9.

109.

James Simpson , A Proposal to Stamp Out Smallpox (Edinburgh: Edmonton and Douglas, 1868).

110.
Reece, *op. cit.* (note 96), 2–3.

111.
Clarke, *op. cit.* (note 70), 458.

112.
‘Smallpox in Liverpool. The Conscientious Objector’s Clause, Suggested Repeal’, *The Liverpool Daily Post*, 31 January (1902), 7.

113.

*Ibid.*, 31 January (1902).

114.

Edward William Hope , Annual Report on the Health of the City of Liverpool during 1903 (Liverpool: C. Tinling and Co., Printing Contractors, 1904), 24.

115.
Albert *et al.*, *op. cit.* (note 59), 375–6.

116.

Hiroshi Nishiura and T. Kashiwagi , ‘Smallpox and Season: Reanalysis of Historical Data’, Interdisciplinary Perspectives in Infectious Disease, 2009 (2009), 591935 doi:10.1155/2009/591935.PMC264866019266090

117.

Sir Leonard Rogers , ‘Smallpox and Climate in England and Wales’, BMJ, 1, February 25 (1928), 300–302.

118.
Liverpool Smallpox Register, *op. cit.* (note 30).

119.

C.T. Ambrose , ‘Osler and the Infected Letter’, Emerging Infectious Diseases, 11, 5 (2005), 689–693.1589012010.3201/eid1105.040616PMC3320382

120.

Philip Boobbyer , ‘Smallpox in Nottingham’, BMJ, 1 (1901), 1054.

121.
Willrich, *op. cit.* (note 39), 276–8.

122.

P.P. Mortimer , ‘Ridding London of Smallpox: The Aerial Transmission Debate and the Evolution of a Precautionary Approach’, Epidemiology and Infection, 136 (2008), 1297–1305.1832512810.1017/S0950268808000459PMC2870728

123.

Derrick Baxby , ‘Studies in Smallpox and Vaccination’, Reviews in Medical Virology, 12, 4 (2002), 201–209.1212501210.1002/rmv.361

124.

*Ibid.*, (note 96).

125.
Edward William Hope, *Observations of the Medical Officer of Health on the Report of Dr R.J. Reece to the Local Government Board on Smallpox and Smallpox Hospitals at Liverpool, 1902–3*, ordered by the Port Sanitary and Hospitals Committee (Liverpool: C. Tinling and Co., 1905).

126.
William Knollys, *On the ‘Observations’ of Dr Hope, Medical Officer of Health for the City of Liverpool, upon the Report of Their Inspector, Dr Reece, on ‘Smallpox and Smallpox Hospitals in Liverpool’, and Also on Dr Hope’s Previous Report on Smallpox in Liverpool*, William Edward Knollys, Assistant Secretary Local Government Board, Whitehall, Addressed to the Town Clerk, Local Government Board Liverpool. London: Whitehall, 11 August 1905.

127.
Mortimer, *op. cit.* (note 122).

128.

*Memorandum by Dr R.J. Reece, as to Questions Raised by Dr Hope Regarding His Report on Smallpox and Smallpox Hospitals at Liverpool 1902–3*, Richard J. Reece, 6 July 1905, https://search.wellcomelibrary.org/iii/ encore/record/C_Rb2136006.

129.
‘Smallpox Case Renews “Move Hospital” Call’, *Bebington News and Advertiser*, 19 April (1958), 1.

130.
‘Disease Spread by Flies Says Ex-Health Chief’, *Bebington News and Advertiser*, 24 May (1958), 5.

131.
‘Residents Irate at Hospital Board Rebuff, Forming Committee of Public Safety’, *Bebington News and Advertiser*, 19 July (1958), 1.

132.
‘New Ferry Smallpox Hospital to Be Moved’, *Bebington News and Advertiser*, 13 September (1958), 1.

133.

*Bebington News and Advertiser*, *op. cit.* (note 130).

134.

P.F. Wehrle , J. Posch , K.H. Richter and D.A. Henderson , ‘An Airborne Outbreak of Smallpox in a German Hospital and Its Significance with Respect to Other Recent Outbreaks in Europe’, Bulletin of the World Health Organisation, 43, 5 (1970), 669–679.PMC24278005313258

135.

David H. Henderson , ‘Smallpox Epidemic Models and Response Strategies’, Biosecurity and Bioterrorism: Biodefense Strategy, Practice, and Science, 11, 1 (2013), 73–74.10.1089/bsp.2013.121123488670

136.

Donald K. Milton , ‘What Was the Primary Mode of Smallpox Transmission? Implications for Biodefense’, Frontiers in Cellular and Infection Microbiology, 2 (2012), 151.2322668610.3389/fcimb.2012.00150PMC3509329

137.
Mark Pallen, *The Last Days of Smallpox, Tragedy in Birmingham* (Independently published, 2018), 230.

138.

Vibha Bhatnagar , Michael A. Stoto , Sally C. Morton , R. Boer and S.A. Bozette , ‘Transmission Patterns of Smallpox: Systematic Review of Natural Outbreaks in Europe and North America Since World War II’, BMC Public Health, 6 (2006), 126, doi:10.1186/1471-2458-6-126.16677388PMC1525174

139.

J. Bincer , H. Wysoczynska and B. Trzaska , ‘Smallpox Epidemic in Gdansk in 1962’, Przegl Epidemiology, 19, 4 (1965), 421–431.5882917

140.

Martin Eichner , ‘Case Isolation and Contact Tracing Can Prevent the Spread of Smallpox’, American Journal of Epidemiology, 158 (2003), 118–128.1285122410.1093/aje/kwg104

141.

F. Fenner , D.A. Henderson , I. Arita I , Z. Ježek and I.D. Ladnyi , Smallpox and Its Eradication. Epidemiology Section (Geneva: World Health Organisation, 1988), 208.

142.

Karl Pearson , ‘On the Criterion That a Given System of Deviations from the Probable in the Case of a Correlated System of Variables is Such That it Can Be Reasonably Supposed to Have Arisen from Random Sampling’, Philosophical Magazine, Series 5, 50 (1900), 157–175.

143.

Major Greenwood and G.U. Yule , ‘The Statistics of Anti-Typhoid Vaccine and Anti-Cholera Inoculations, and the Interpretation of Such Statistics in General’, Proceedings of the Royal Society of Medicine (Section Epidemiology and Statistics Medicine), 8 (1915), 113–194.10.1177/003591571500801433PMC200418119978918

144.

Edward J. Edwardes , ‘A Century of Vaccination: Smallpox Epidemics and Smallpox Mortality Before and Since Vaccination Came into Use’, BMJ, 2 (1902), 27–30.2076034810.1136/bmj.2.2166.27PMC2401272

145.

W.R. MacDonnell , ‘On the Influence of Previous Vaccination in Cases of Small-pox’, Biometrika, 1, 3 (1902), 375–383.

146.

A. Rusnock , ‘Medical Statistics and Hospital Medicine: The Case of the Smallpox Vaccination’, Centaurus, 49 (2007), 337–359.1854346210.1111/j.1600-0498.2007.00084.x

147.

James Colgrove , ‘Between Persuasion and Compulsion: Smallpox Control in Brooklyn and New York, 1894–1902’, Bulletin of the History of Medicine, 78 (2004), 349–378.1521105210.1353/bhm.2004.0062

148.
Hopkins, *op. cit.* (note 2), 293.

149.

Mrs Garrett Anderson , ‘On the Administration of the Laws for the Prevention of Epidemic Smallpox’, BMJ, 2 (1902), 30–32.2076034910.1136/bmj.2.2166.30PMC2401263

150.

Nadja Durbach , Bodily Matters. The Anti-vaccination Movement in England, 1853–1907 (Durham, NC: Duke University Press, 2005), 10.

151.

E.P. Hennock , ‘Vaccination Policy Against Smallpox, 1835–1914: A Comparison of England with Prussia and Imperial Germany’, Social History of Medicine, 11 (1998), 49–71.1162015410.1093/shm/11.1.49

152.
Albert, *op. cit.* (note 59), and ’Hearing Over’, *The Boston Globe*, 5 February (1902), 4.

153.
Wallach, *op. cit.* (note 11), 6–7, 102–26, 146–62. Wallach outlines the 1902 campaign to amend the compulsory vaccination laws and the conflict between the anti-vaccinationists and Dr Samual Durgin, Chair of Public Health. The Massachusetts state legislature went back and forth on the issue of compulsory vaccination for several years, but in 1914 amended the law to allow unvaccinated children admission to public schools if their parents opposed vaccination (Wallach, 216).

154.

*Ibid.,*116–20.

155.

W.E. Parmet , R.A. Goodman and Farber, ‘Individual Rights Versus the Public’s Health – 100 Years after *Jacobson v. Massachusetts*
’, New England Journal of Medicine, 352, 7 (2005), 652–654.1571655810.1056/NEJMp048209

156.
The background and history of this judicial court activity are discussed in detail in Wallach, *op. cit.* (note 11) chapter nine, *Jacobson versus Massachusetts, 187–214*.

157.

Dorothy Porter and Roy Porter , ‘The Politics of Prevention: Anti-vaccinationism and Public Health in Nineteenth-Century England’, Medical History, 32 (1988), 231–252.306390310.1017/s0025727300048225PMC1139881

158.
Wallach, *op. cit.* (note 11), 84–8.

159.
‘New Stations Opened on Huntington Ave.’, *The Boston Globe*, 19 November 1901; ‘Lull in Vaccination’, *The Boston Globe*, 8 December (1901); ‘Dr A.W. Bowman Takes Issue with Other Physicians and the Board of Health’, *The Cambridge Chronicle*, 9 August (1902), 6.

160.
‘Smallpox at New Ferry, Reassuring Statement by Dr Kenyon’, *The Birkenhead and Cheshire Advertiser and Wallasey Guardian*, 1 February (1902), 8.

161.

Edward J. Edwardes , A Concise History of Smallpox and Vaccination in Europe (London: Lewis, 1902), 133.

162.

*The Liverpool Daily Post*, *op. cit.* (note 112).

163.
‘Is Vaccination Efficient? Value and Defects’, *The Liverpool Daily Post*, 1 February, (1902), 6.

164.
Hennock, *op. cit.* (note 151).

165.

H. Nishiura , K. Dietz and M. Eichner , ‘The Earliest Notes on the Reproduction Number in Relation to Herd Immunity: Theophil Lotz and Smallpox Vaccination’, Journal of Theoretical Biology, 241 (2006), 964–967.1687589210.1016/j.jtbi.2006.01.012

166.

Clarissa Damaso , ‘Revisiting Jenner’s Mysteries, the Role of the Beaugency Lymph in the Evolutionary Path of Ancient Smallpox Vaccines’, Lancet Infectious Diseases, 18 (2018), e55–e63.2882714410.1016/S1473-3099(17)30445-0

167.
Williams, *op. cit.* (note 42), 270.

168.

Livia Schrick , Clarisso Damaso , José Esparza and Andreas Nitsche , ‘An Early American Smallpox Vaccine Based on Horsepox’, The New England Journal of Medicine, 377 (2017), 15–16.10.1056/NEJMc170760029020595

169.
M.J. Rosenaau, ‘The Bacteriological Impurities of Vaccine Virus. An Experimental Study’, *Hygienic Laboratory Bulletin No. 12, March 1903. Public Health and Marine-Hospital Service of the United States* (Washington: Government Printing Office, 1903).

170.
Walter Reed was a United States army physician who won fame in 1901 leading the team that postulated and confirmed the theory that yellow fever is transmitted by a particular mosquito species, rather than by direct contact.

171.
Willrich, *op. cit.* (note 39), 184.

172.

Editorial, ‘Government Control of Vaccine Virus’, Journal of the American Medical Association, 43, 26(1904), 1974.

173.

*Ibid.*, 1974.

174.
Hanna, *op. cit.* (note 32), 24.

175.

Hiroshi Nishiura , Stefan O. Brockman and Martin Eichner , ‘Extracting Key Information from Historical Data to Quantify the Transmission Dynamics of Smallpox’, Theoretical Biology and Medical Modelling, 5 (2008), 20.1871550910.1186/1742-4682-5-20PMC2538509

176.
Eichner, *op. cit.* (note 94), 719–21.

177.

William Hanna , Studies in Smallpox and Vaccination (London: Wright and Sons, 1913).

178.

*Studies in Smallpox and Sanitation Based on the Experience of the City of Liverpool. A Reply to the Book Entitled* Studies in Smallpox and Vaccination *by William Hanna* (The National Anti-Vaccination League, Strand, London, 1913).

179.
In the Annual Report for 1885 the then Medical Officer of Health, Dr J. Stopford Taylor, estimated that 92% of the people of Liverpool were more or less efficiently vaccinated. His successor, Dr E.W. Hope, said that there were grounds for supposing that Liverpool was amongst the best vaccinated communities in the country.

180.

*The Liverpool Daily Post*, *op. cit.* (note 112).

181.
‘Smallpox in Liverpool, Traceable to Original Source’, *The Liverpool Daily Post*, 5 February (1902), 4.

182.

George Bernard Shaw , ‘Mr Bernard Shaw on Smallpox Prevention’, BMJ, 2 (1901), 1289–1291.20759858PMC2507338

183.

*The Liverpool Echo*, Advertisement, 16 January (1902), 3.

184.

*The Boston Globe*, *op. cit.* (note 77).

185.
’Anti-vaccination’, *The Cambridge Chronicle*, 29 March (1901).

186.
Albert, *op. cit.* (note 59), 378, Figure 3.

187.

*The Boston Post*, Radam’s microbe killer advertisment, 4 December (1901), 7.

188.

*The Cambridge Chronicle*, Lifebuoy soap advertisement, 28 June (1902), 3.

189.
Wallach, *op. cit.* (note 11), 84.

190.
This episode is outlined in Wallach, *op. cit.* (note 11): Immanuel Pfeiffer versus the Boston Board of Health, 127–45.

191.
McVail, *op. cit.* (note 49), 24.

192.
Dixon, *op. cit.* (note 67), 314.

193.
Port Sanitary Hospital, City of Liverpool Smallpox Register, *op. cit.* (note 30).

194.

Thomas M. Mack , ‘Smallpox in Europe, 1950–1971’, The Journal of Infectious Disease, 125 (1972), 161–169.10.1093/infdis/125.2.1615007552

195.

Alexander S.M. Macgregor , Report of the Public Health Department (Glasgow, 1942).

196.
Dixon, *op. cit.* (note 67), 341.

197.

J. Pickford Marsden , ‘Ship-borne Smallpox’, BMJ, 2 (1942), 706.

198.
Dixon, *op. cit.* (note 67), 342.

199.

Derrick Tovey , ‘The Bradford Smallpox Outbreak in 1962: A Personal Account’, Journal of the Royal Society of Medicine, 97 (2004), 244–247.1512181910.1258/jrsm.97.5.244PMC1079469

200.
Chapin, *op. cit.* (note 3), 190.

201.
Willrich, *op. cit.* (note 39), 338–9.

202.

F. Guerra , ‘The European-American Exchange’, History and Philosophy of the Life Sciences, 15, 3 (1993), 313–327.7529930

203.

P.D. Curtin , ‘Disease Exchange across the Tropical Atlantic’, History and Philosophy of the Life Sciences, 15, 3 (1993), 329–356.7529931

204.

George A. Babbitt , ‘Retirement of Dr Samuel H. Durgin from the Boston Health Board’, American Journal of Public Health (New York), 2, 5 (1912), 384–385.10.2105/ajph.2.5.384PMC108937618008672

205.
Henderson, *op. cit.* (note 135).

206.

M.S. Keckler , M.G. Reynolds , I.K. Damon and K.L. Karem , ‘The Effects of Post-Exposure Smallpox Vaccination on Clinical Disease Presentation: Addressing the Data Gaps between Historical Epidemiology and Modern Surrogate Model Data’, Vaccine, 31 (2013), 5192–5201.2399437810.1016/j.vaccine.2013.08.039PMC4623313

207.
Edward William Hope, *Health at the Gateway: Problems and International Obligations of a Seaport City* (Cambridge, MA: Cambridge University Press, 1931). Biographical details in: June Clayton, ‘Aspects of the Life and Work of Dr. Edward William Hope, Assistant Medical Officer of Health for Liverpool, 1883–94, and Medical Officer of Health for Liverpool, 1894–1924’ (Unpublished MPhil thesis: University of Liverpool, 1999).www.evolve360.co.uk/Data/10/Docs/13/13Clayton.pdf.

208.
McVail, *op. cit.* (note 49), 24.

209.
Hopkins, *op. cit.* (note 2), 291.

210.
Chapin, *op. cit.* (note 3), 194.

